# Unconventional function of an Achaete-Scute homolog as a terminal selector of nociceptive neuron identity

**DOI:** 10.1371/journal.pbio.2004979

**Published:** 2018-04-19

**Authors:** Neda Masoudi, Saeed Tavazoie, Lori Glenwinkel, Leesun Ryu, Kyuhyung Kim, Oliver Hobert

**Affiliations:** 1 Department of Biological Sciences, Howard Hughes Medical Institute, Columbia University, New York, United States of America; 2 Department of Systems Biology, Columbia University Medical Center, New York, United States of America; 3 Department of Brain and Cognitive Sciences, DGIST, Daegu, South Korea; New York University, United States of America

## Abstract

Proneural genes are among the most early-acting genes in nervous system development, instructing blast cells to commit to a neuronal fate. *Drosophila* Atonal and Achaete-Scute complex (AS-C) genes, as well as their vertebrate orthologs, are basic helix-loop-helix (bHLH) transcription factors with such proneural activity. We show here that a *C*. *elegans* AS-C homolog, *hlh-4*, functions in a fundamentally different manner. In the embryonic, larval, and adult nervous systems, *hlh-4* is expressed exclusively in a single nociceptive neuron class, ADL, and its expression in ADL is maintained via transcriptional autoregulation throughout the life of the animal. However, in *hlh-4* null mutants, the ADL neuron is generated and still appears neuronal in overall morphology and expression of panneuronal and pansensory features. Rather than acting as a proneural gene, we find that *hlh-4* is required for the ADL neuron to function properly, to adopt its correct morphology, to express its unusually large repertoire of olfactory receptor–encoding genes, and to express other known features of terminal ADL identity, including neurotransmitter phenotype, neuropeptides, ion channels, and electrical synapse proteins. *hlh-4* is sufficient to induce ADL identity features upon ectopic expression in other neuron types. The expression of ADL terminal identity features is directly controlled by HLH-4 via a phylogenetically conserved E-box motif, which, through bioinformatic analysis, we find to constitute a predictive feature of ADL-expressed terminal identity markers. The lineage that produces the ADL neuron was previously shown to require the conventional, transient proneural activity of another AS-C homolog, *hlh-14*, demonstrating sequential activities of distinct AS-C-type bHLH genes in neuronal specification. Taken together, we have defined here an unconventional function of an AS-C-type bHLH gene as a terminal selector of neuronal identity and we speculate that such function could be reflective of an ancestral function of an “ur-” bHLH gene.

## Author summary

Across the animal kingdom, transcription factors of the basic helix-loop-helix (bHLH) family act during embryonic nervous system patterning as proneural genes to promote neuroblast identity. We describe here a distinct function for a specific member of this family, *hlh-4*, in the nematode *Caenorhabditis elegans*. *hlh-4* is exclusively expressed in a nociceptive neuron class and is not required for this neuron class to be generated but is rather required for the execution of its terminal differentiation program. *hlh-4* directly controls the expression of scores of terminal identity features of this neuron class, including its large battery of chemoreceptor-encoding genes. We propose that a role of bHLH genes in controlling terminal differentiation may be the ancestral function of members of this gene family.

## Introduction

Nervous system development proceeds through sequential steps, starting with the early commitment to a neuronal fate, followed by the progressive restriction of fates, to finally reaching a terminal, differentiated end state. Proneural genes of the basic helix-loop-helix (bHLH) family play a key role in the initial stages of this process [1]. Mutant analysis in *Drosophila* revealed that loss of members of the Achaete-Scute complex (AS-C), as well as the related Atonal gene, resulted in the loss of the ability to generate neuroblasts in the peripheral nervous system [2–5]. Vertebrate orthologs of proneural AS-C and Atonal genes (the Mash and Math genes) also provide critical proneural function in vertebrate nervous system development [1,6–8]. Thus, the proneural function of AS-C-type and Atonal bHLH genes is broadly conserved throughout evolution.

The *C*. *elegans* genome encodes a canonical complement of homologs of proneural bHLH genes, including seven AS-C-like genes (*hlh-4*, *hlh-3*, *hlh-14*, *hlh-19/hnd-1*, *hlh-12*, *hlh-6*, *hlh-16*) and one Atonal ortholog (*lin-32*) [9]. The function of many of these *C*. *elegans* bHLH genes in the nervous system has not been as extensively studied as their fly and vertebrate orthologs, but it is nevertheless clear that a number of these bHLH genes also provide proneural activities [10–12]. Like in flies and vertebrates, *C*. *elegans* proneural bHLH genes operate in a lineage-specific manner. For example, the *C*. *elegans* AS-C ortholog *hlh-14* and the *C*. *elegans* Atonal ortholog, *lin-32*, provide proneural activity in several distinct sensory neuron lineages of the peripheral and central nervous system (CNS) of the worm [10–12]. In both cases, the proneural activity of *hlh-14* and *lin-32* is exemplified by a transformation of neuroblasts into cells with a hypodermal identity in the respective mutant backgrounds.

One question that has been studied extensively over the years is whether AS-C/Atonal-type bHLH genes have functions in the nervous system that go beyond their proneural activity. In both vertebrates and flies, nonproneural functions of AS-C and Atonal-like genes have indeed been described in the context of later neuronal differentiation events (reviewed in [1,6,13]). Similarly, *C*. *elegans lin-32/*Ato has functions beyond its proneural activity in male ray lineages in which *lin-32* also allocates fates in subsequently developing ray sublineages [14]. However, in all these cases, the respective bHLH gene is either transiently expressed; acts through downstream, intermediary regulatory factors; or only affects selected aspects of the differentiated state of the respective neuron.

In this study, we describe a novel, nonproneural, and noncanonical function of an AS-C-type bHLH gene. We find that the AS-C homolog *hlh-4* displays a spatial and temporal specificity of expression that is unprecedented for any bHLH gene. *hlh-4* is exclusively and continuously expressed in a single postmitotic nociceptive sensory neuron class in which it initiates and maintains the terminal identity of this neuron via direct binding to scores of terminal effector genes that are expressed in a neuron class–specific manner and that define the differentiated state of this neuron. Among its many functions in ADL, *hlh-4* directly regulates the expression of the unusually large repertoire of olfactory receptor proteins in ADL. We hypothesize that the direct control of “neuron function genes” may have been an ancestral function of bHLH genes.

## Materials and methods

### Strains

Strains were maintained by standard methods [15]. A list of all strains used is listed in **S3 Table**.

### Expression constructs and transgenic strain generation

Green fluorescent protein (GFP) reporters for rescue and ectopic expression were generated using RF-cloning [16]. For making G-protein coupled receptor (GPCR) transgenic reporters (listed in S3 Table), a PCR fusion approach was used [17]. Genomic fragments were fused to the GFP coding sequence, which was followed by the *unc-54* 3′ untranslated region. All transgenic lines created in this study were injected at 50 ng/μL with the *unc-122*::*gfp* into wild-type animals or with the *pha-1* rescuing plasmid (pBX) as a coinjection marker (50 ng/μL) into *pha-1* mutant animals. For each construct, two independent lines were scored.

Fosmid-based reporters for *hlh-2*, *hlh-3*, and *hlh-4* were generated by insertion of *yfp* at the 5′ end of the *hlh-2* locus [18], 3′ end of *hlh-4* (this paper), and *gfp* at the 3′ end of *hlh-3* [19] using standard fosmid recombineering approaches [19,20].

The *arrd-4* promoter (1,587 bp) was cloned together with *hlh-4* genomic sequences and *unc-54* 3′UTR into a pPD95.75 backbone and injected (50 ng/μL) into OH14884 as a simple array, with *unc-122*::*gfp* (50 ng/μL) as a coinjection marker. The *unc-3* promoter fusion was generated by amplification of 558 bp of *unc-3* promoter, fused to *hlh-4* genomic (including its own 3′UTR), using the PCR fusion approach [17]. Fifty nanograms per milliliter of this construct were injected into OH14884, with *ttx-3*::*mcherry* as a coinjection marker.

The *eat-4* reporter constructs were generated by PCR and subcloning into pPD95.75 vector. *eat-4prom6-1* contains 4,450 bp of the upstream region of the ATG and *eat-4prom2* contains 1,150 bp of the genomic region just upstream of the ATG. The E-Box and homeodomain motif are found at positions -693 and -726 relative to the ATG start codon, respectively. The specific sequences deleted are, for the E-Box, AACAGGTGTT, and for the homeodomain site, ATTAGATAAT. The deletions were generated by mutagenesis with the QuickChange Site-Directed Mutagenesis kit (Stratagene). The plasmids were injected into OH13645 *[otIs518;him-5(e1490)]* at 50 ng/μL, using *unc-122*::*gfp* (50 ng/μL) as a coinjection marker.

### Microscopy

Worms were anesthetized using 50 mM sodium azide (NaN_3_) and mounted on 5% agarose on glass slides. Images were acquired using an automated fluorescence microscope (Zeiss, AXIO Imager Z.2) or LCS-8 laser point scanning confocal. Representative images are shown following maximum projection of Z-stacks using the maximum intensity projection type. Image reconstruction was performed using Fiji software [21].

### Neuron identification

ADL neurons were identified by labeling subsets of sensory neurons with DiD or DiO (Thermo Fisher Scientific). For dye filling, worms were washed with M9 and incubated at room temperature with DiD (1:500) in M9 for 1 hour for Adults or (1:250) for 2 hours for L1 stage animals. After incubation, worms were washed three times with M9 and plated on agar plates coated with food (OP50 bacteria) for 1–3 hours before imaging.

### Embryonic expression pattern analysis

The expression of bHLH fosmid reporters was manually lineaged using SIMI BioCell program, as previously described [22]. Briefly, the gravid adults of *hlh-4*^*Fosmid*^::*yfp (otIs683)* and *hlh-3*^*fosmid*^::*gfp (otIs648)* were dissected and single two-cell embryos were mounted and visualized on a Zeiss Imager Z1 compound microscope using the 4D microscopy software, Steuerprg (Caenotec). Nomarski stacks were taken every 30 seconds and embryos were illuminated with LED fluorescence light (470 nm) at predetermined time points during development.

### Avoidance assay

Avoidance assay was performed as previously described [23,24]. L4 stage animals were picked onto OP50 seeded plates before a day of assay. We used 100 nM or 500 nM ascr#3 or 1M glycerol diluted in M13 buffer. In the assay, M13 buffer was firstly dropped in front of animals’ heads. When the animals didn’t respond to M13 buffer, we then dropped ascr#3/glycerol and checked avoidance to the stimulus. Long reversals were counted as avoidance [25]. The tests were done at least 5 times with 10 animals each.

### DNA motif discovery

Motif discovery was carried out using information-theoretic analysis as implemented in the Finding Informative Regulatory Elements (FIRE) algorithm [26]. De novo motifs were discovered by running FIRE in discrete mode, with all the genes in the *C*. *elegans* genome labeled as either belonging to class 1: the neuron-specific expression class (e.g., 117 ADL-expressed genes) or class 2: the complementary set of all other remaining genes. The starting *k*-mer seed length was set to *k* = 6 and the sequence search space was confined to 2-kb upstream regions. The discovered CACCTG motif had a robustness score of 10/10 with a significance z-score of 18.3.

### Phylogenetic footprinting

We used TargetOrtho [27] to find whole genome CACCTG motif matches in five nematode genomes searching 2 kb upstream of each gene plus introns. ADL-expressed genes and all *C*. *elegans* genes, excluding noncoding RNAs, were compared using the Wilcoxon rank sums test to assess alignment independent species conservation scores, motif match position relative to the start codon, and motif match frequency per gene. Only genes with at least one CACCTG match were analyzed.

## Results

### *hlh-4* is exclusively expressed in nociceptive ADL neurons

**Fig 1:**
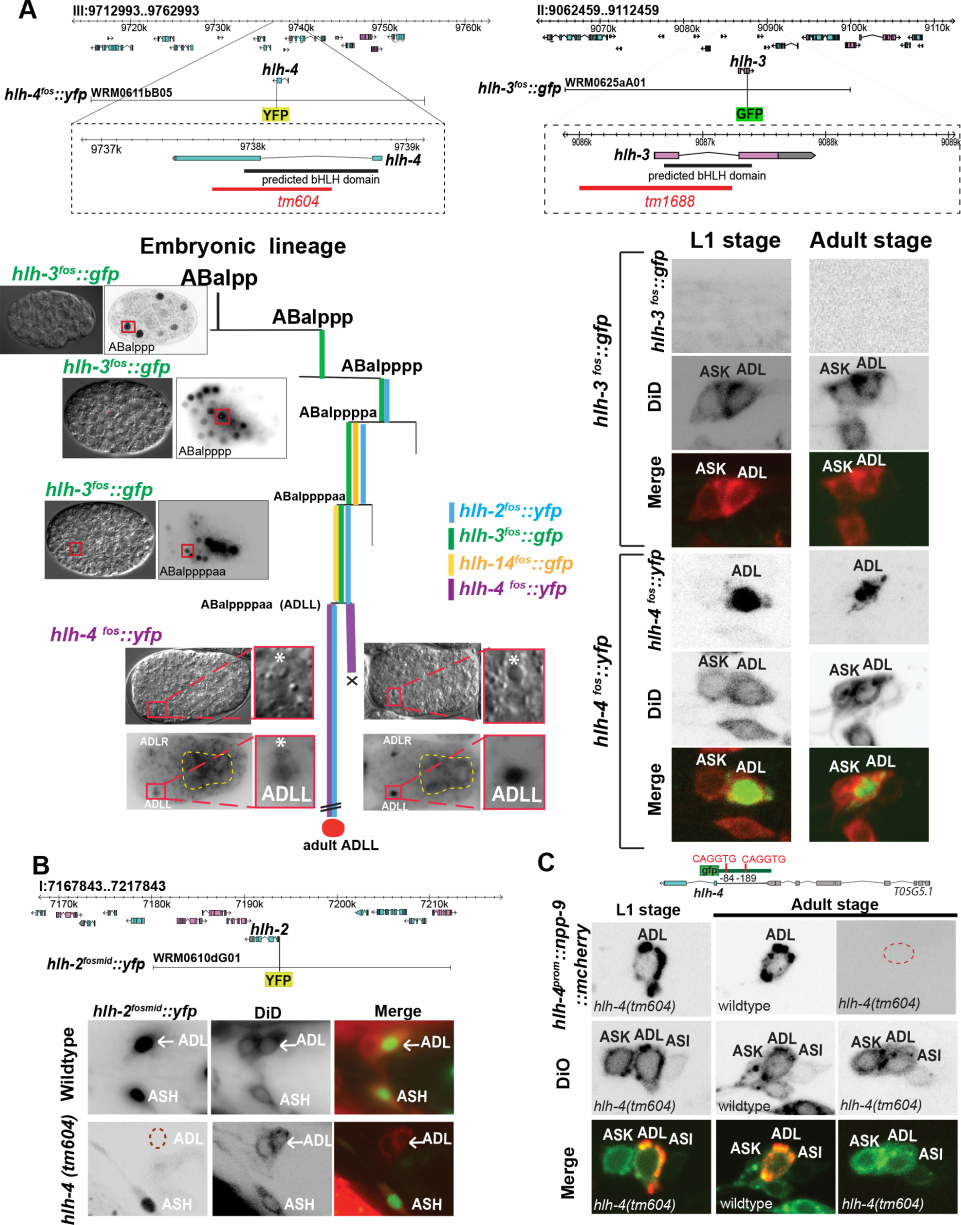
***hlh-4* and *hlh-2* expression in postmitotic ADL neurons. (A)** *hlh-4* and *hlh-3* fosmid reporters and their expression patterns. Schematic of gene structure shows that the deletion in *tm604* and *tm1688* alleles removes a major part of the bHLH domain from both genes. Lineage diagram showing the specific cells from ADL ancestors that express *hlh-2*, *hlh-3*, *hlh-4*, and *hlh-14* during embryogenesis. Representative images of *hlh-3* and *hlh-4* gene expression at embryonic stages next to their exact time point during embryonic development (left). *hlh-4* fosmid reporter is first detected in ADLs and their sister cells as soon as they are born (left panel). Roughly 30 minutes after they are born, sisters of ADLs die by apoptosis (Asterisks indicate ADLs sister cells that are destined to die by apoptosis) and *hlh-4* expression becomes restricted to ADLs only (right panel). Yellow dashed line is marking gut autofluorescent. Expression of *hlh-2* in the dying ADL sister could not be examined. *hlh-14* expression is shown for comparison and was reported in [12]. **(B)** Schematic of fosmid reporter for *hlh-2*. HLH-2 is continuously expressed in very few neurons throughout adulthood, among them ADL, and this continuous ADL expression depends on *hlh-4*. Previous work had examined expression of *hlh-2* in L1 stage animals only [29]. **(C)** Continuous *hlh-4* expression in ADL is assured via autoregulation. In *hlh-4* mutants, *hlh-4* expression, as assessed with a *hlh-4* promoter fusion, initiates normally at the embryonic stage; however, it fails to maintain the expression past L1 stage. bHLH, basic helix-loop-helix; L1, first larval stage.

As a first step toward a systematic analysis of the neurogenic function of *C*. *elegans* bHLH genes, we undertook a nervous system–wide expression pattern analysis of all *C*. *elegans* AS-C-like genes. Using fosmid-based reporter transgenes, we found that many bHLH genes are expressed during embryonic development within and outside neuronal lineages, but we noticed that one AS-C-like bHLH gene, *hlh-4*, displays an unusual expression pattern, both in terms of spatial and temporal specificity (**Fig 1**). *hlh-4* expression is not observed in any blast cells during embryonic or postembryonic development but rather is first expressed in two pairs of postmitotic cells in the precomma stage embryo, shortly after their birth (**Fig 1A**). One pair is the ADL neurons and the other pair is the sisters of ADL, which die shortly after their birth by programmed cell death [28]. Expression of *hlh-4* in ADL is observed for the remainder of embryogenesis, continues during larval and adult stages, and is never observed in any other cell throughout the entire organism (**Fig 1A**). The fosmid on which the *yfp* reporter construct is based is able to fully rescue the *hlh-4* mutant phenotype that we describe below (rescue data are shown in **Table 1**). The ADL-specific fosmid-based reporter expression pattern is recapitulated by a 700-bp 5′ promoter fusion reporter (**Fig 1C**).

**Table 1:** Rescue of the *hlh-4* mutant phenotype. *srh-127*::*gfp* expression was expressed from the *otIs646* array. The *hlh-4* fosmid is WRM0611bB05.

Genotype	Transgene	*srh-127*::*gfp* in ADL (% animals)	*n*
wild type	none	100%	>100
*hlh-4* *(tm604)*	none	0%	32
	*otEx4130[hlh-4*^*fosmid*^::*yfp; ttx-3*::*mcherry]*	100%	25
	*otEx7180[hlh-4* ^*fosmid*^*; ttx-3*::*mcherry]*, line #1	100%	27
	*otEx7181[hlh-4*^*fosmid*^*; ttx-3*::*cherry]*, line #2	100%	25
	*otEx7179[arrd-4*^*prom*^::*HLH-4*::*rfp; unc-122*::*gfp]*	100%	41

With the exception of *hlh-3*, which is expressed in a subclass of postmitotic motor neurons of the ventral nerve cord [31], none of the other *C*. *elegans* AS-C-like bHLH genes (*hlh-6*, *hlh-12*, *hlh-14*, *hlh-16*, *hlh-19/hnd-1*) share the postmitotic, post-developmental neuronal expression feature of *hlh-4* [12,32–34]. We note that while our fosmid-based *hlh-3* reporter showed extensive expression in blast cells during embryogenesis, it does not recapitulate the postembryonic ADL expression previously reported using a reporter that only contained 1.5 kb of 5′ sequences upstream of the gene [35].

The only other bHLH reporter expressed in postmitotic neurons throughout embryonic, larval, and adult stages is the Daughterless homolog *hlh-2/*Da [29], a binding partner of many *C*. *elegans* AS-C-related bHLH genes [30]. Expression of HLH-2/DA protein in a specific subset of postmitotic neurons, including the nociceptive neurons ADL and ASH, has been previously reported using anti-HLH-2 antibody staining [29], but it was not reported whether expression persisted into later larval and/or adult stage. Using a fosmid-based reporter of *hlh-2/*Da expression, we found that ADL and ASH expression of *hlh-2/*Da, as well as expression in a few other head and tail neurons, is maintained throughout all larval stages into adulthood (**Fig 1B**). We conclude that *hlh-4/AS-C* and its heterodimerization partner *hlh-2/*Da are continuously coexpressed specifically in the nociceptive ADL neuron class.

### Continuous *hlh-4* and *hlh-2* expression is ensured by transcriptional autoregulation

One well-documented mechanism by which transcription factors ensure their continuous expression throughout the life of a neuron is through transcriptional autoregulation (e.g., [36–39]). To assess whether continuous expression of *hlh-4* throughout the life of the ADL neuron is also ensured by autoregulation, we used a 5′ promoter fusion of the *hlh-4* locus, which recapitulated the continuous expression of *hlh-4* in ADL (**Fig 1C**). We crossed this reporter into an *hlh-4* mutant allele, *tm604*, a putative null allele generated by the *C*. *elegans* knockout consortium in Tokyo [40] in which the bHLH domain is largely deleted (**Fig 1A**). We found that *hlh-4* reporter expression in the ADL neuron pair is initiated normally in *hlh-4* mutant embryos, but expression fails to be maintained beyond the first larval stage (**Fig 1C**). As yet unknown factors may initiate *hlh-4* expression in the embryo and, after its initiation, *hlh-4* takes over to regulate its own expression.

We furthermore tested whether continuous expression *hlh-2/*Da in ADL requires *hlh-4* activity. Crossing the *hlh-2* fosmid reporter into the *hlh-4* mutant background, we indeed found this to be the case (**Fig 1B**). We conclude that the continuous expression of both *hlh-4* and its putative cofactor *hlh-2/*Da is based on transcriptional autoregulation.

### *hlh-4* does not act as a proneural gene

**Fig 2:**
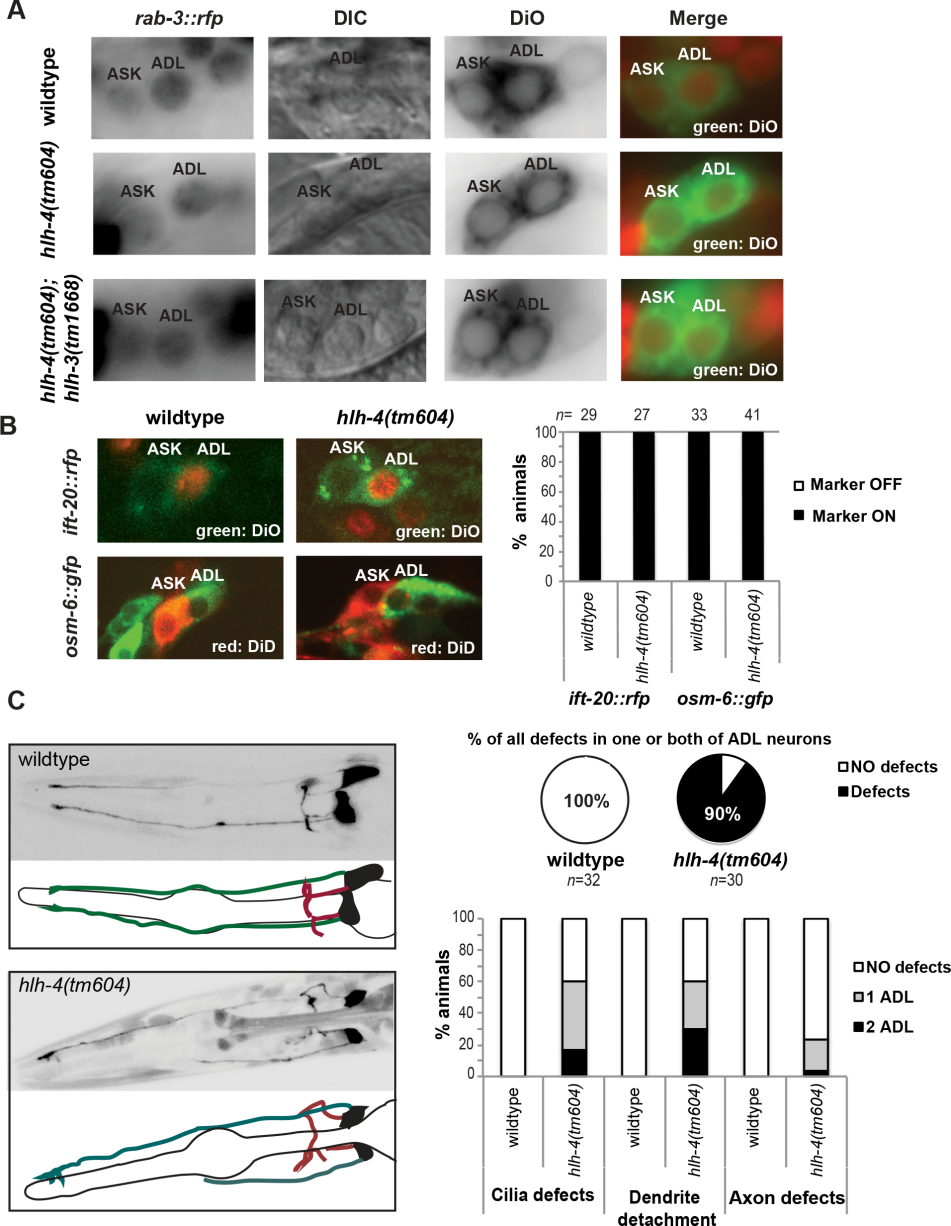
*hlh-4* does not operate as a proneural gene to control generation of the ADL nociceptive neurons but affects its morphological differentiation. **(A)** Expression of panneuronal gene *rab-3* is unaffected in ADL neurons of *hlh-4* mutants. Moreover, ADL neurons exhibit stereotypic speckled neuronal nuclei (shown here by Nomarski) and display normal dye filling ability in *hlh-4* single null mutants and also in *hlh-3; hlh-4* double null mutants. **(B)** The pansensory identity of ADL neurons in *hlh-4* mutant is intact, shown here using ciliated genes markers (*osm-6* and *ift-20*). Numerical values that underlie the graph are also shown in S1 Data. **(C)** *hlh*-4 mutants display ADL neuron morphology defects. ADL is visualized with a *hlh-4prom* reporter whose expression is still visible at the first larval stage (as shown) of *hlh-4* mutants. Defects include (1) detachment of dendrites (labeled in green) from the nose (surprisingly, even the detached ADL neurons are still able to dye fill, as inferred by the completely unaffected dye filling of *hlh-4* mutants [panel A]), (2) cilia defect (no branching or extra branching of normally bifurcated ciliated ending), and (3) axon (labeled in red) branching defect. Numerical values that underlie the graph are shown in S1 Data.

In most if not all organisms examined, AS-C genes have proneural function, characterized by a loss of neuroblast identity in the absence of the AS-C gene and ensuing conversion into an ectodermal identity [1,3,6,13]. Previous work has demonstrated that in the lineage that produces ADL, as well as other sensory neurons, the transiently and early-expressed AS-C gene *hlh-14* acts as a proneural gene, such that loss of *hlh-14* results in a neuroblast to hypodermal fate conversion [12]. In striking contrast, we find that the later-expressed *hlh-4* gene does not act as a proneural gene. Specifically, in *hlh-4* null mutants, the ADL neuron pair is still generated and differentiates as a neuron, as assessed by (a) intact expression of a panneuronal reporter, *rab-3*, (b) intact filling of the ADL neuron with the dye DiI (which is taken up by the dendritic endings of several sensory neurons, including ADL [41]), and (c) presence and intact speckled appearance of the ADL neuronal nucleus by Nomarski optics (**Fig 2A**). Corroborating this notion, we find that the two genes that are expressed by all ciliated sensory neurons, *osm-6* and *ift-20* [42,43], are still normally expressed in the ADL neurons of *hlh-4* mutants (**Fig 2B**). Even though we could not confirm the previously reported expression of *hlh-3* in ADL (**Fig 1A**), we nevertheless generated *hlh-3; hlh-4* double null mutants and found that in these animals the ADL neurons are also still generated normally, as assessed by intact DiI filling and characteristic neuronal nuclear speckles (**Fig 2A**).

The expression of the *hlh-4* promoter fusion in *hlh-4* mutants until the first larval stage permitted us to visualize the anatomy of the ADL neurons in the absence of *hlh-4* gene function. While the cell body of ADL is normally positioned, we find that ADL axons and dendrites display severe morphological defects (**Fig 2C**). The sensory dendrites of ADL are often detached from the nose. Even when attached, the cilia of ADL often do not display their characteristic bifurcated ciliated endings. The axons of ADL, which in wild-type animals display a highly stereotyped extension and branching pattern, show pathfinding and branching defects (**Fig 2C**).

### *hlh-4* affects expression of the unusually large repertoire of olfactory receptors in ADL

**Fig 3:**
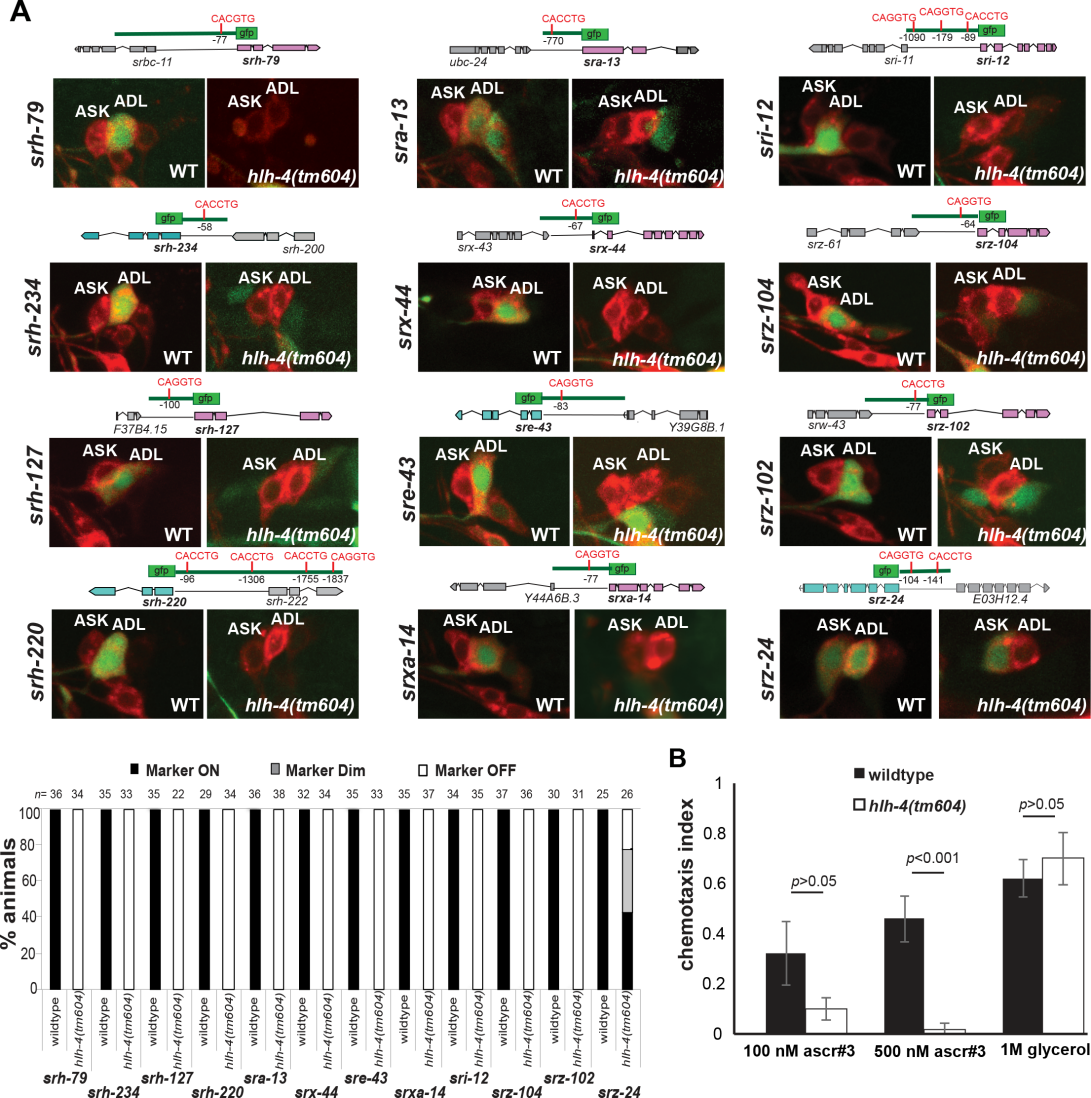
*hlh-4* is required for chemoreceptor expression and chemosensory function of the ADL neurons. **(A)** Effect of *hlh-4(tm604)* allele on GPCR reporter expression. DiD staining (red) is used to label the amphid neurons, including ADL. Numerical values that underlie the graph are shown in S1 Data. **(B)** ADL-mediated chemosensory behavior toward C9 ascaroside. Statistical significances shown were calculated with the one-way ANOVA with Dunnett’s test. Numerical values that underlie the graph are shown in S1 Data. WT, wild-type.

To examine whether and to what extent *hlh-4* is required to specify ADL neuron identity, we examined the differentiation program of the ADL neurons in detail. The ADL nociceptive neuron pair coexpresses an unusually large number of olfactory-type GPCRs [44–46]. Reporter genes generated for about one fifth of the approximately 1,300 GPCR encoding reveal the expression of more than 60 GPCR genes from diverse families in ADL [46]. Extrapolating to the complete set of GPCRs encoded in the *C. elegans* genome, about 300 GPCR-encoding genes may be expressed in ADL. We asked whether *hlh-4* is required for the expression of 12 GPCR-encoding genes. We chose these genes to cover the diverse set of GPCR gene families expressed in ADL (*sra*, *sre*, *sri*, *srz*, *srh*, *srxa*, and *srx* families). We found that expression of all of the tested 12 GPCR reporters is abrogated in *hlh-4* mutants (**Fig 3A**). While all defects were routinely scored at the adult stage, we note that these defects are already apparent at the first larval stage. Consistent with the absence of expression of the *hlh-4* paralog *hlh-3* in postmitotic ADL neurons, we find that *hlh-3* does not affect *srh-127* expression in ADL.

To test whether *hlh-4* does not only affect expression of chemoreceptor proteins but also affects the chemorepulsive function mediated by the ADL neurons, we considered its chemorepulsive function toward a specific nematode pheromone, the ascaroside ascr#3 (asc-ΔC9, C9)[24]. While wild-type hermaphrodites are repelled by ascr#3, this repulsion is significantly reduced in *hlh-4* hermaphrodites (**Fig 3B**). This is not a reflection of an overall failure to engage in a nociceptive response because another chemorepulsive behavior, mediated by the ASH neurons (glycerol avoidance) [47], is not affected in *hlh-4* mutants (**Fig 3B**).

### *hlh-4* specifies the neuron type–specific molecular signature of ADL

**Fig 4:**
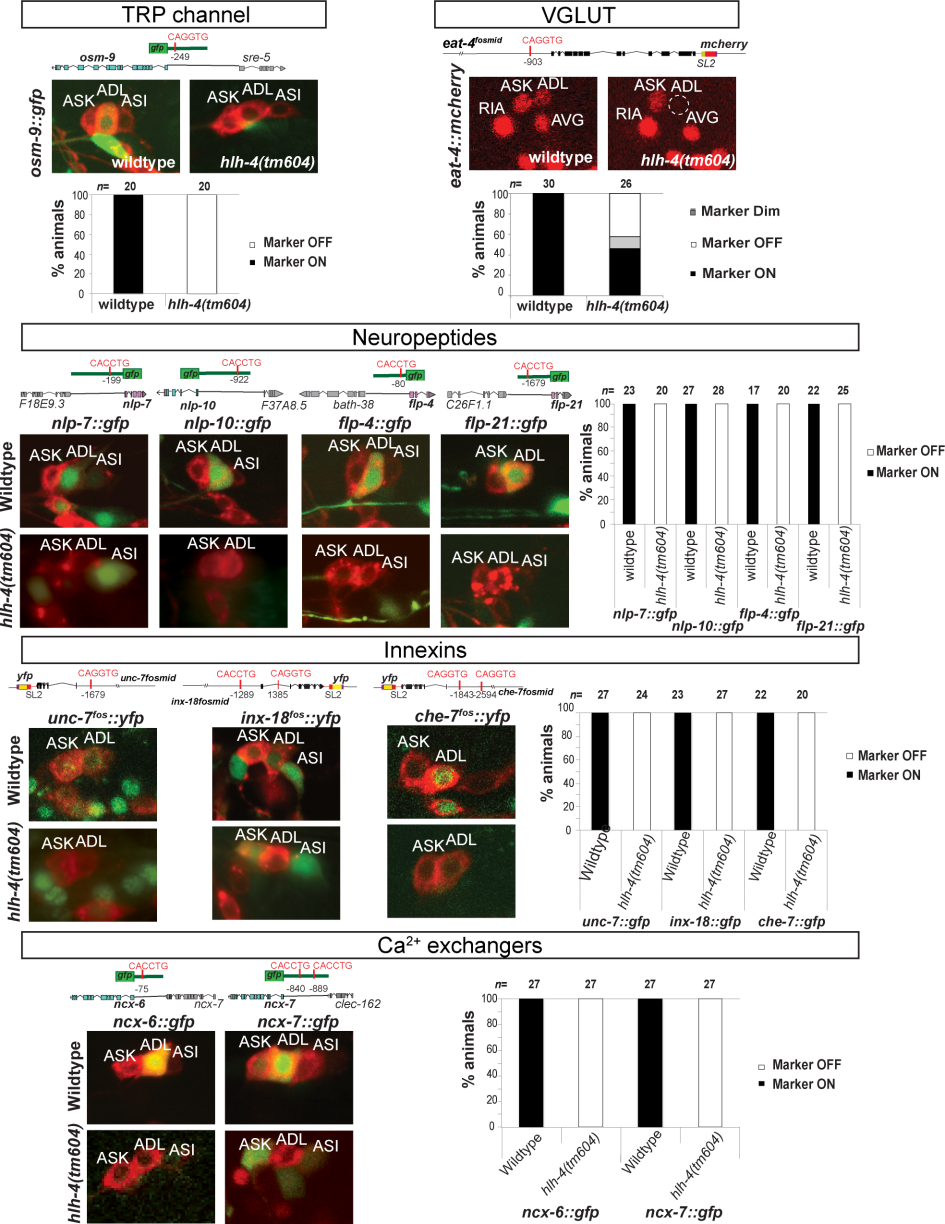
*hlh-4* is required for the acquisition of terminal ADL identity. Indicated *gfp* reporters were crossed into *hlh-4(tm604)* mutants and expression quantified. The function/identity of the marker genes is indicated above each panel. In all panels in which a *gfp* reporter is used, DiD staining (red) is used to label the amphid neurons, including ADL. Numerical values that underlie the graph are shown in S1 Data.

We tested whether *hlh-4* function is restricted to controlling olfactory receptor expression and function in the ADL neurons or whether other identity features of ADL are disrupted as well. A TRP channel protein encoded by the *osm-9* gene, expressed in a restricted set of sensory neurons, is required in ADL to signal the response to distinct chemorepulsive sensory inputs [24,48,49]. We find that *osm-9* expression is selectively lost in the ADL neurons of *hlh-4* mutant animals (**Fig 4**).

Going beyond signal perception and transmission, we asked whether ADL requires *hlh-4* to communicate with its synaptically connected neurons [50]. Based on the expression of the vesicular glutamate transporter *eat-4/VGLUT*, the key defining feature of all glutamatergic neurons, ADL neurons have previously inferred to be glutamatergic [51]. We find that the glutamatergic identity of ADL, as assessed by *eat-4* fosmid reporter gene expression, is defective in *hlh-4* mutant animals (**Fig 4**). Apart from using glutamate as a likely fast neurotransmitter, the expression patterns of various neuropeptide-encoding genes indicate that ADL also utilizes distinct peptides for neurotransmission [52,53]. We find that the expression of four neuropeptides, previously known to be expressed in ADL, as well as other neurons (FMRFamides *flp-4* and *flp-21* and neuropeptides *nlp-7* and *nlp-10*) [52,53] specifically fail to be expressed in the ADL neurons of *hlh-4* mutants, while expression in other neurons is unaffected (**Fig 4**).

Apart from peptidergic and chemical synaptic transmission, electrical synaptic transmission is likely also affected in *hlh-4* mutants. ADL forms electrical synapses with a select number of neighboring neurons [50]. Electrical synapses are formed by transmembrane innexin proteins [54], and 3 of the 24 *C*. *elegans* innexin genes, *unc-7*, *inx-18*, and *che-7*, are expressed in ADL, as well as a specific set of other neuron types [55]. The expression of all three innexin genes is lost specifically in the ADL neurons of *hlh-4* mutants (**Fig 4**).

Transmembrane ion channel expression is also affected in *hlh-4* mutants. Na^+^/Ca^2+^-K^+^ exchangers are important regulators of intracellular calcium homeostasis in the nervous system, and members of this family show remarkably specific gene expression profiles in the *C*. *elegans* nervous system [56]. Two Na^+^/Ca^2+^-K^+^ exchangers, *ncx-6* and *ncx-7*, are each exclusively expressed in the ADL neurons of wild-type animals [56]. The expression of both genes in ADL is abrogated in *hlh-4* mutants (**Fig 4**).

To examine whether these defects are a consequence of the failure of solely maintaining the differentiated state versus failure of initiation of the differentiated state, we examined the expression of several ADL markers right after *hlh-4* mutant embryos had hatched. Testing four specific markers (*srh-127*, *sre-43*, *srt-47*, and *ncx-6*), we found that expression is already affected at this early stage of development.

In conclusion, we find that several distinct identity features that define functional features of the ADL neuron are coregulated by the same transcription factor. The affected identity features share the common theme of providing the ADL with a unique molecular signature and identity. In contrast, *hlh-4* does not affect generic neuronal features (i.e., pansensory or panneuronal features).

### *hlh-4* is sufficient to induce ADL features in other neuron classes

**Fig 5:**
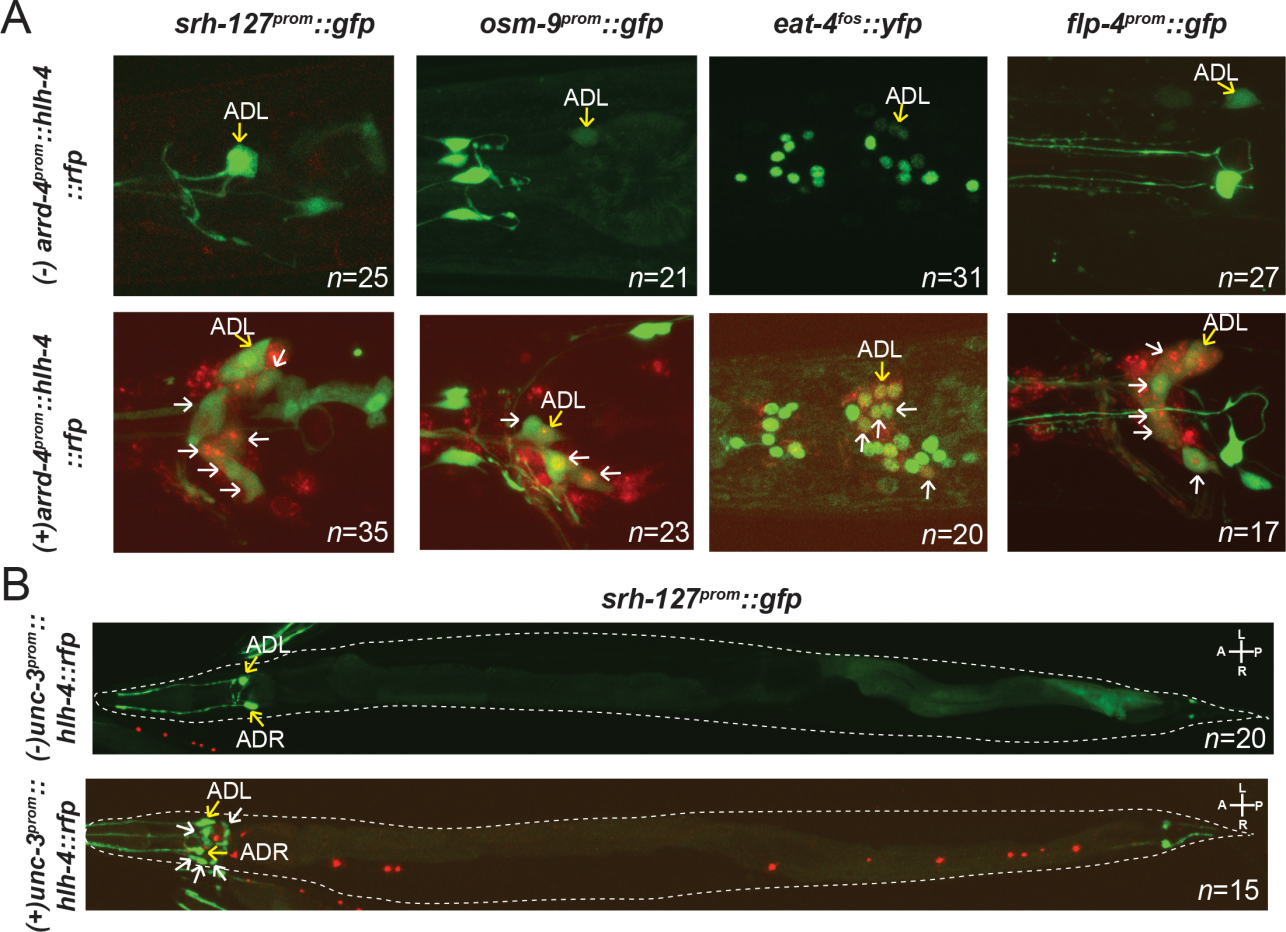
*hlh-4* is sufficient to induce ADL marker in other chemosensory neurons. **(A)** Transgenic animals ectopically expressing *hlh-4* with the pan-cilia-promoter *arrd-4*, and the effect on chromosomally integrated reporters for ADL identity, *srh-127*, *osm-9*, *eat-4*, *and flp-4*. In the lower panels, white arrows indicate the ectopic cells that are now induced by HLH-4 to express ADL-fate markers. Yellow arrows mark ADL neurons. The ectopic marker expression effect is fully penetrant and numbers of animals scored are shown. **(B)** Transgenic animals ectopically expressing *hlh-4* under control of a promoter fragment from the *unc-3* locus. The effect on the chromosomally integrated reporters for ADL identity, *srh-127*, is shown. This promoter fragment of the *unc-3* locus recapitulates *unc-3* expression in ventral nerve cord motor neurons and, ectopically, in unidentified head neurons (**S1 Fig**). When driving *hlh-4*, ADL marker expression is induced in head neurons but not in the ventral nerve cord. The ectopic marker expression effect is fully penetrant and can be detected in approximately four extra neurons, in the head (marked with white arrows). The numbers of animals scored are shown.

*hlh-4* is not only required for the expression of ADL identity genes, but ectopic expression of *hlh-4* is also sufficient to induce ADL identity features. We drew this conclusion by driving expression of *hlh-4* in many other ciliated sensory neurons, using the *arrd-4* promoter [57] (**S1 Fig**). The *arrd-4prom*::*hlh-4* construct is not only able to rescue the loss of *srh-127*::*gfp* expression in ADL in *hlh-4* mutants (**Table 1)**, but these transgenic animals display ectopic expression of the normally ADL-expressed *srh-127*::*gfp* reporter in many ciliated sensory neurons (**Fig 5A**). Similarly, the TRP channel *osm-9*, the neuropeptide-encoding *flp-4* gene and the vesicular glutamate transporter *eat-4* also are ectopically expressed in other sensory neurons in these transgenic animals (**Fig 5A**).

To further probe the ability of *hlh-4* to induce ADL identity features in other neurons, we misexpressed *hlh-4* under control of a promoter fragment from the *unc-3* locus, which is expressed in ventral cord motor neurons and a small set of head neurons (**S1B Fig**). Transgenic animals expressing a *unc-3prom*::*hlh-4* construct show ectopic expression of the ADL marker *srh-127*::*gfp* in head neurons but not in ventral cord motor neurons (**Fig 5B**). The apparent cellular context dependency of *hlh-4* function mimics the context dependence of other master regulators of cellular identity, such as Eyeless/Pax6 [58].

### *cis*-Regulatory regions of ADL-expressed genes are enriched for a specific E-box motif

**Fig 6:**
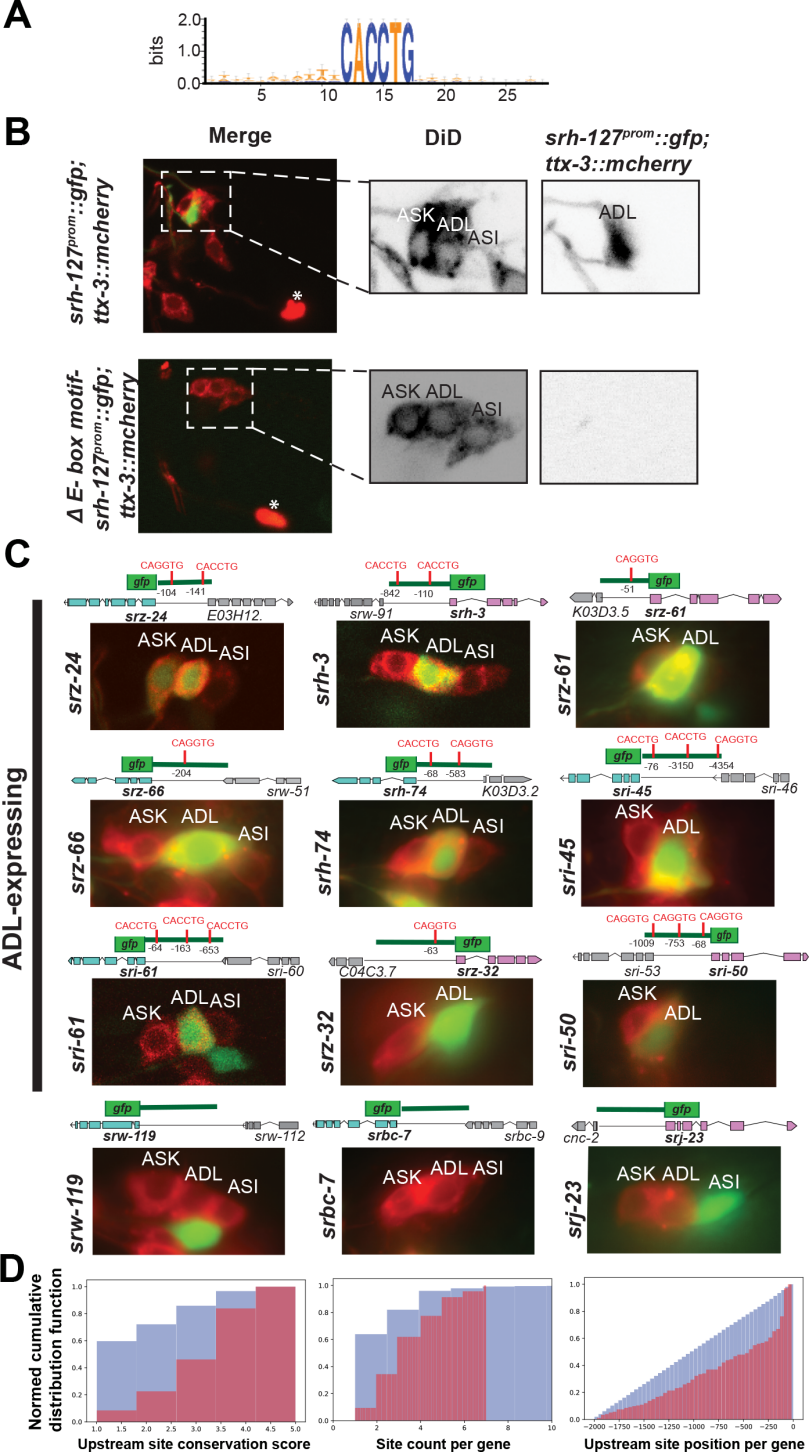
The HLH-4/HLH-2 E-box motif is required for ADL expression and is a predictor for ADL expression. **(A)** Motif logo representation of the E-box of ADL-expressed genes. **(B)** Deletion of E-box motif in the promoter of *srh-127*, a GPCR that normally expresses in ADL, abolishes the ADL expression. Asterisks are marking *ttx-3*::*mcherry* expression, used as a coinjection marker. **(C)** Reporter expression pattern of GPCRs that contain or do not contain the indicated E-box core motif CACCTG. DiD staining (red) is used to label the amphid neurons, including ADL. **(D)** Cumulative distributions of *Caenorhabditis elegans* upstream CACCTG conservation per gene (left), CACCTG site count per gene (middle), and CACCTG upstream site position per gene (right). Blue: whole genome genes; purple: ADL-expressed genes. ADL genes had motif matches that were conserved in 3.39 species’ orthologs, on average, versus 1.85 species’ orthologs amongst all genes with at least one CACCTG site match (Wilcoxon test statistic = 11.34, *p* = 8.23e−30). ADL genes had 3.23 CACCTG matches per gene compared to 2.67 CACCTG matches genome wide per gene with at least one site match (Wilcoxon test statistic = 4.41, *p* = 9.90e−06). CACCTG site positions in ADL-expressed genes were on average 657.35 bases upstream of the start codon compared to 1,001.77 bases upstream of the start codon genome wide per gene with at least one site match (Wilcoxon test statistic = 7.98, *p* = 1.42e−15).

Because gene expression is usually examined in *C*. *elegans* via reporter gene constructs, a large library of reporter transgenes that monitors the expression of thousands of genes has been amassed by the *C*. *elegans* community over the past few decades. In many cases, expression patterns of these reporter transgenes have been defined on a single neuron level. Almost 200 reporter transgenes have been found to be expressed in the ADL neurons (www.wormbase.org, **S2 Table**). The genes tested above for their dependence on *hlh-4* belong to this dataset. We took a subset of these genes (117) and asked whether 5′ upstream regulatory regions of genes whose expression is monitored by these reporter transgenes are enriched for the presence of a specific sequence motif using the FIRE motif analysis platform [26] (see Materials and methods). We restricted the search space to the first 2 kb upstream of these genes. As a control, we also considered several other neuron classes that Wormbase associated with a large number of reporter genes (AIY, ASE, ALM, HSN, ASI, ASK, ASH, PHA; www.wormbase.org) and interrogated the upstream regulatory control regions of those genes. In the ADL dataset, we indeed identified a motif found in 75% of the ADL-expressed reporter genes **(Table 2, S1 Table; S2 Table)**. The motif, shown in **Fig 6A**, has a completely invariant 6-nucleotide core, CACCTG, and no striking sequence features outside this core. There is no orientation preference for this motif on the plus versus minus strand. This motif is not enriched in the control datasets (AIY, ASE, ALM, HSN, ASI, ASK, ASH, or PHA expressed reporter genes).

**Table 2:** ADL-expressed effector genes, presence of E-box, and *hlh-4* dependence. For more detail in genes and for precise location of the motif see S1 Table. For primary data see Fig 2 and Fig 3.

Category	presence of CACCTG E-box	*hlh-4* dependent
sensory receptors (GPCR and rGCY)	68/73	12/12 tested
GPCR trafficking	3/3	
neurotransmitter (Glu, neuropeptides)	6/6	5/5 tested
neurotransmitter receptors	3/5	
transporter	2/3	2/2 tested
channels	6/7	1/1 tested
electrical synapse (innexins)	5/5	3/3 tested
cytoskeleton	2/2	
transmembrane/adhesion	2/2	
small secreted peptide	2/2	
signaling/enzymes	8/11	
novel	1/3	
**Total**	**108/122**	**23/23**

Abbreviations: Glu, glutamate; GPCR, G-protein coupled receptor; rGCY, receptor-type guanylyl cyclase.

The CACCTG motif matches experimentally determined bHLH binding sites (CANNTG) [59] and specifically matches the in vitro binding site of the *C*. *elegans* HLH-4/HLH-2 heterodimer, CA(G/C)CTG [30]. Probabilistic segmentation analysis of upstream regulatory sequences of ADL neuron-expressed GPCR genes had previously also identified a similar CA(G/C)CTG motif [45].

All the 23 terminal effector genes that we described above as depending on *hlh-4* in their expression in ADL (**Fig 3**; **Fig 4**) contain at least one copy of this motif within 2 kb upstream of the 5′ start of the gene (**Table 2, S1 Table**). The one *hlh-4*-dependent GPCR reporter (*srh-79*) that does not contain a perfect match to the E-box motif contains a 1-nucleotide-mismatched copy of the motif (CACGTG versus CACCTG).

The *hlh-4* locus itself and, specifically, the 700-bp 5′ upstream regulatory region that shows *hlh-4* autoregulation (**Fig 1C**) contains two copies of the perfectly matched CACCGT motif (both motifs are located in the 245-bp-long intergenic region). Moreover, the upstream region of the *hlh-2/*Da gene, the putative cofactor of *hlh-4*, which is also continuously expressed in ADL, also contains three copies of this motif in its 5′ upstream intergenic region. The regulation of *hlh-2/*Da expression by *hlh-4* (demonstrated above) is therefore also likely a reflection of direct autoregulation of the *hlh-2* locus by the HLH-4/HLH-2 heterodimer.

Three lines of evidence further validate the importance of the CACCGT E-box motif for ADL expression:

1. We mutated the CACCGT E-box motif in one of the newly discovered, *hlh-4-*dependent targets, *srh-127*, and found that this mutation abolished expression in ADL (**Fig 6B**).
2. We examined whether a set of 35 ADL-expressed reporter genes not included as a training set for the FIRE analysis also contain the CACCTG motif. All of these 35 reporter genes code for GPCRs that were found to be expressed in ADL after the initial FIRE analysis was performed [46]. We found that 33 out of the 35 ADL-expressed reporters contain the CACCTG motif (**Table 2, S1 Table**). In contrast to the presence of the E-box motif in ADL-expressed and *hlh-4-*dependent genes, we found that panneuronal genes [60] are largely devoid of the CACCTG E-box (*rab-3*, *ric-4*, *snb-1*, *unc-64*, *sng-1*, *unc-10*, *unc-18*, *snn-1*, *egl-3*, *and egl-21* do not contain an E-box within 2 kb of their start sites, while *unc-11* and *snt-1* do).
3. We generated 12 reporters to additional sets of genes (again all GCPR-encoding genes) that the FIRE analysis revealed to either contain or not contain this motif. All of the nine genes that contain a CACCTG motif indeed showed expression in ADL (**Fig 6C**). Three GPCR reporters that do not contain a CACCTG motif show no expression in ADL (**Fig 6C**).

### Phylogenetic conservation of the E-box motif and further validation of its importance for ADL expression

We used phylogenetic footprinting in the TargetOrtho pipeline [27] to assess the extent of conservation of the CACCTG motif among five *Caenorhabditis* species, *C*. *elegans*, *C*. *briggsae*, *C*. *remanei*, *C*. *brenneri*, and *C*. *japonica* (**S2 Table**). This analysis provided a genome-wide assessment of the location of the CACCTG motif in these five different species and allowed us to define a number of features of the CACCTG motif:

1. The ADL-expressed genes tend to have more conserved CACCTG motifs among phylogenetically conserved, orthologous genes compared to any gene in the genome that contains a CACCTG motif (**Fig 6D**).
2. ADL genes have more CACCTG motifs compared to any gene with a CACCTG in the genome. This is true for all *Caenorhabditis* species but is most obvious in *C*. *elegans* (**Fig 6D**).
3. The upstream CACCTG positions are closer to the start codon in the known ADL-expressed genes compared to any gene with a CACCTG in the genome (**Fig 6D**). This trend is most obvious in *C*. *elegans* but is also significant in the other four *Caenorhabditis* species.

Moreover, we find that two of the ADL-expressed genes that do not contain a perfect match to the CACCTG motif (*srh-79* and *srh-186*, one of which, *srh-79*, we confirmed to be *hlh-4*-dependent) contain a motif with a single mismatch to the CACCTG motif (CACGTG), yet all *Caenorhabditis* species that have orthologues of these two genes contain perfect CACCTG motif matches (**Table 2, S1 Table**).

In conclusion, a CACCTG motif defines a signature for ADL-expressed genes. Given that this motif is a known in vitro binding site for a HLH-4/HLH-2 dimer [30], *hlh-4* appears the most likely candidate to directly activate the expression of scores of genes that uniquely and combinatorially define the terminally differentiated state of the ADL neuron pair.

### *hlh-4* displays complex regulatory interactions with the *lin-11* LIM homeobox gene

**Fig 7:**
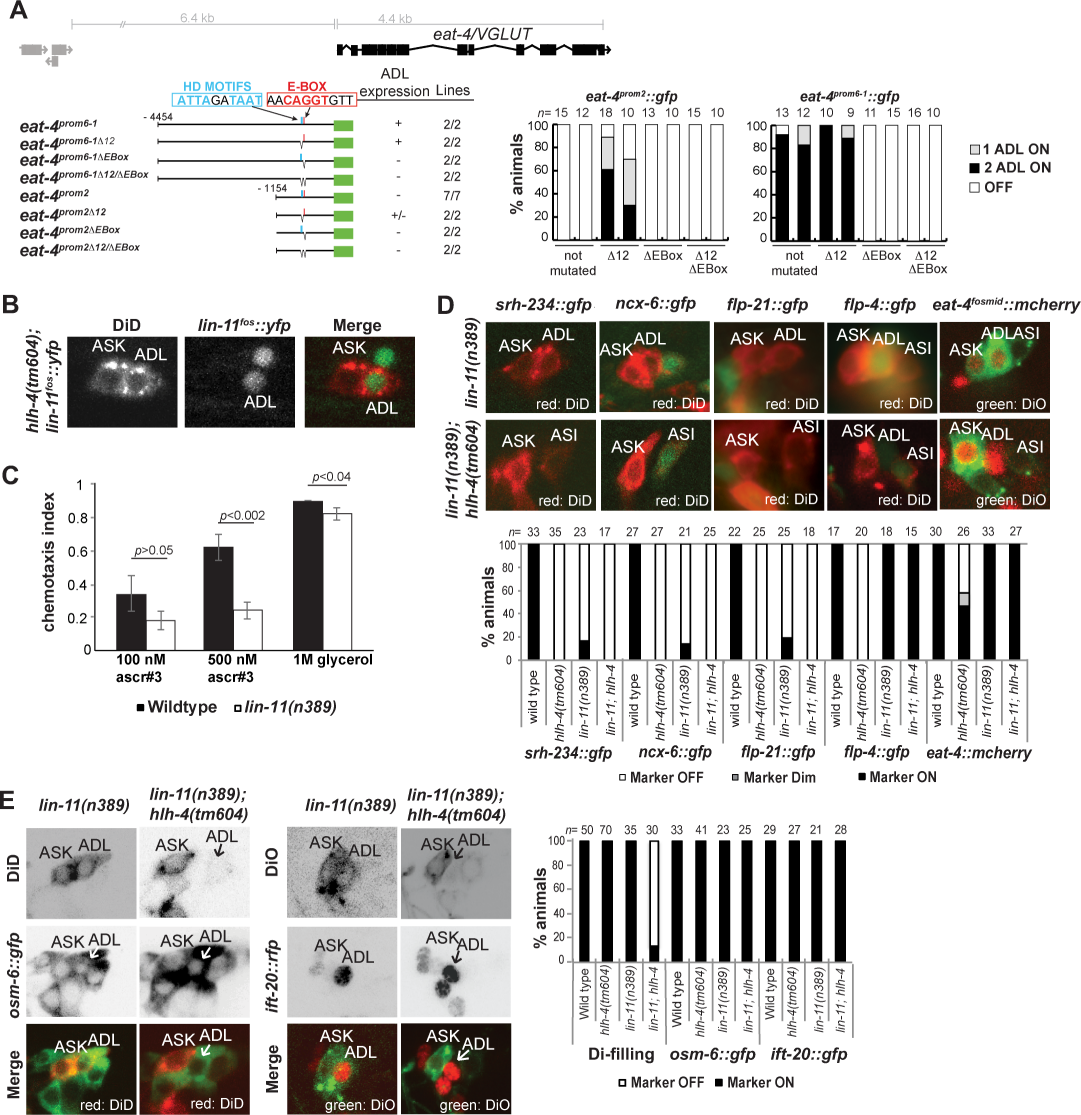
*lin-11* also contributes to ADL differentiation. **(A)** Analysis of the *cis*-Regulatory control region of the *eat-4* locus. The precise nature of the deletion of the motifs is shown in the Materials and methods section. Scoring of expression of two lines is shown in the right panel. It was previously published that an integrant of the transgene *eat-4prom2* was expressed in ADL (*otIs376*) [51]. We have since analyzed multiple extrachromosomal lines of *eat-4prom2* (seven lines all showing similar expression; quantification of two lines is shown here) as well as two lines of *eat-4prom1* (a slightly larger construct; not shown) and found none of these lines to display expression in ADL. The previously reported expression of these transgenes in ADL was likely an array artifact that affected the repressive effect of the homeodomain binding sites described here. **(B)** *lin-11* fosmid reporter expression is not affected in *hlh-4* null mutants. **(C)** ADL-mediated chemosensory behavior toward C9 ascaroside. Statistical significances shown were calculated with the one-way ANOVA Dunnett’s test. **(D)** Effect of *lin-11* null mutants on terminal ADL markers, alone or in combination with *hlh-4* null mutants. The previously reported partial effect of *lin-11* on the brightness of expression of an *eat-4* fosmid (*otIs388*) [51] could not be repeated with this or other *eat-4* reporter transgenes. The data from *hlh-4* and N2 are repeated (from Figs 3A, 4) in the graph for ease of comparison. **(E)** ADL neurons of *lin-11; hlh-4* mutants fail to dye fill but are still generated as assessed by expression of pansensory marker. For ease of comparison the data from *hlh-4* and N2 are repeated (from Fig 2B) in the graph. Numerical values that underlie the graph shown in this figure are shown in S1 Data.

The partially penetrant effect of *hlh-4* on *eat-4/VGLUT* expression suggested that *hlh-4* partly relies on additional factors to control *eat-4/VGLUT* expression. This notion is further corroborated through an examination of the *cis-*Regulatory control regions of the *eat-4/VGLUT* locus. We find that 4.5 kb of sequence upstream of the *eat-4/VGLUT* locus directs reporter gene expression to many glutamatergic neurons, including ADL (prom6-1; **Fig 7A**). This 4.5-kb region contains a phylogenetically conserved CACCTG motif 691 bp upstream of the ATG. Deletion of this motif results in loss of expression in ADL (**Fig 7A**). However, while this motif is required for ADL expression, it is apparently not sufficient: deleting 3.2 kb from the 4.5-kb 5′ reporter fusion leaves the E-box unaffected but abolishes expression in ADL (prom2; **Fig 7A**), suggesting that these deleted sequences contain binding site(s) for a transcription factor that cooperates with *hlh-4* to activate *eat-4/VGLUT* expression.

The LIM homeobox gene *lin-11* was previously shown to be expressed in postmitotic ADL neurons throughout their lifetime [61]. We find that *lin-11* expression in ADL is not affected in *hlh-4* mutants (**Fig 7B**). Corroborating a role of *lin-11* in parallel to *hlh-4*, we find that *lin-11* null mutants are defective in the ADL-mediated chemorepulsive response to C9 ascaroside (**Fig 7C**). Consistent with this behavioral defect, we observed that *lin-11* null mutants display defects in the expression of several of *hlh-4*-dependent and E-box-containing genes, including *ncx-6*, *srh-234*, and *flp-21* (**Fig 7D**). However, *lin-11* does not affect the *hlh-4-*dependent *flp-4* gene, nor does it affect *eat-4/VGLUT* fosmid reporter expression (**Fig 7D).**

We tested whether a function for *lin-11* on *eat-4/VGLUT* expression could be revealed in the context of an *hlh-4* mutant background, in which *eat-4/VGLUT* fosmid reporter expression is only partially affected. *lin-11; hlh-4* double mutants still normally express pansensory markers in ADL, but they display a dye filling defect that neither mutant alone displays, corroborating the parallel nature by which *hlh-4* and *lin-11* affect ADL differentiation (**Fig 7E**). Surprisingly, in *hlh-4; lin-11* double null mutants, the partially penetrant loss of *eat-4/VGLUT* expression observed in *hlh-4* single mutants was not enhanced but instead completely suppressed (**Fig 7D**). The same effect is observed on the *flp-4* gene. Its completely penetrant loss in *hlh-4* mutants is suppressed in *hlh-4; lin-11* double mutants (**Fig 7D**).

The reinstatement of *eat-4/VGLUT* fosmid expression even in the absence of *hlh-4* is mirrored by a mutation in the *cis-*Regulatory control region of *eat-4/VGLUT*. The 1.2-kb upstream region of *eat-4/VGLUT*, which contains an *hlh-4* binding site but is not expressed in ADL, becomes expressed in ADL upon deletion of a predicted homeodomain binding site, a potential recognition motif for LIN-11 (**Fig 7A**). This result suggests that *eat-4/VGLUT* expression is controlled via a collaboration of *hlh-4* with an as yet unknown transcription factor X whose activating effect is normally antagonized by LIN-11. If all activators (*hlh-4* and X) are present, *lin-11* cannot prevent activation of *eat-4/VGLUT (eat-4prom6-1delta12)*; hence, *eat-4/VGLUT* is expressed in ADL. If, however, the system is partially destabilized by *hlh-4* removal (or by removal of the E-box sequence in the reporter construct), *lin-11* can counteract the ability of factor X to activate *eat-4/VGLUT* expression (*eat-4prom2delta 12*) (as assessed by the restoration of *eat-4* expression upon removal of *lin-11*). The effect of *lin-11* on ADL-expressed genes is, however, clearly target gene dependent. While in the case of one target gene, *eat-4/VGLUT*, *lin-11* appears to antagonize *hlh-4* function, it may positively cooperate with *hlh-4* on those other target genes whose expression is either completely or partially lost in *hlh-4* and/or *lin-11* mutants. We conclude that *hlh-4* is a central regulator of ADL identity that may interact in a target gene–dependent manner with distinct collaborating factors.

## Discussion

**Fig 8:**
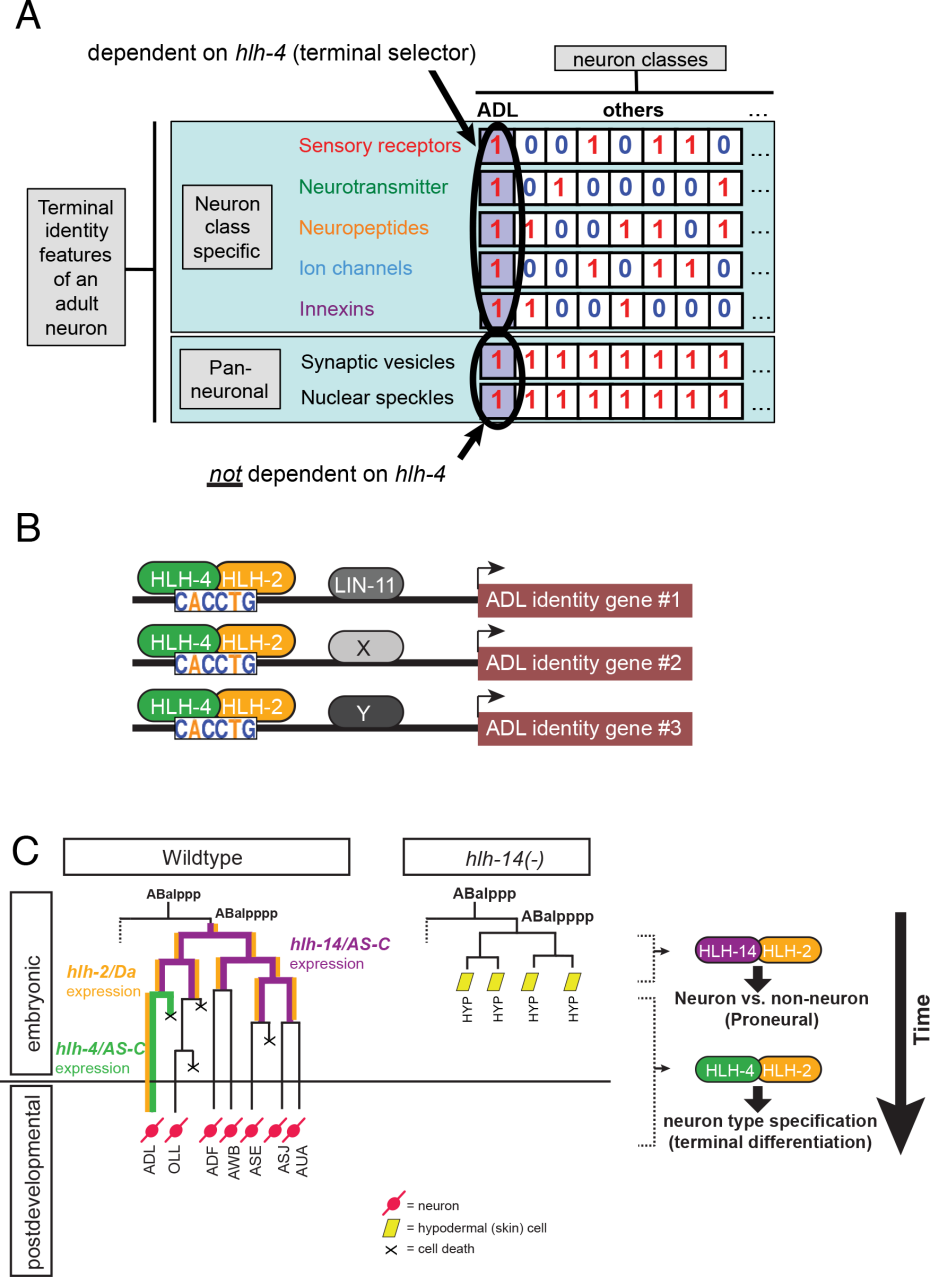
Schematized *hlh-4* functions. **(A)** Overall logic of *hlh-4/*terminal selector function. “0” indicates gene/feature not expressed; “1” indicates expressed (this binary scheme is a simplification). Like other terminal selectors, *hlh-4* genetically separates the adoption of neuron-specific features, i.e., genes expressed in specific parts of the nervous system from the adoption of a panneuronal identity. Rather than being defined by genes uniquely expressed in ADL, ADL identity is uniquely defined by a combinatorial signature of genes expressed in multiple neuron types. If those genes show selective expression in other neuron types, they are terminal selector dependent. **(B)** While a HLH-4/HLH-2 complex appears to be a central regulator of ADL identity genes, it operates with distinct cofactor(s) in a target gene–dependent manner. This is inferred from the notion that *lin-11* acts in parallel to HLH-4/2 to contribute to the activation of some but not other target genes. **(C)** Sequential activities of AS-C-type bHLH genes in a sensory neuron–producing lineage. Lineage diagrams and *hlh-14* data taken from [12]. Note the difference between early *hlh-14* and late *hlh-4* function. Transiently expressed *hlh-14* controls the decision of neuroblast versus ectodermal (hypodermis/skin) while *hlh-4* controls which type of neuronal identity ADL will adopt, through regulation of the ADL-specific molecular signature shown schematically in panel B. AS-C, Achaete-Scute complex; bHLH, basic helix-loop-helix; Da, Daughterless.

The identification of proneural genes that act very early in neuronal development to allocate neuroblast identity to distinct neuronal lineages via classic genetic loss of function analysis in *Drosophila* represents one of the classic landmark achievements of developmental neurogenetics [2,3]. The subsequent cloning of vertebrate AS-C and Atonal homologs has revealed the deep conservation of this fundamental neural patterning mechanism [1,6–8]. We have described here a novel functional property of an AS-C gene, demonstrating that *C*. *elegans hlh-4* joins the rank of terminal selector-type transcription factors that act in postmitotic neuron classes to initiate and maintain the differentiated state of a specific, postmitotic neuron class. *hlh-4* displays all the hallmarks of a terminal selector [62,63]: it is required for initiation of the terminal differentiation program of the ADL neuron pair, it is continually expressed throughout the life of the neuron (suggesting that it also maintains neuronal identity), this continuous expression is mediated by direct autoregulation via HLH-2/HLH-4 binding sites in the *hlh-2* and *hlh-4* loci, and, most importantly, *hlh-4* controls the vast majority of neuron class–specific genes whose combinatorial coexpression defines ADL identity, yet it does not control generic neuronal features (panneuronal and pansensory features). Hence, exactly like other terminal selectors [62,63], *hlh-4* separates the adoption of neuron type–specific features (*hlh-4-*dependent) from the acquisition of an overall, panneuronal/pansensory identity (*hlh-4-*independent) (**Fig 8A**). It is important to precisely appreciate this fundamental dichotomy in neuronal gene expression programs, repeatedly observed in many different neuron classes and corroborated here by the *hlh-4* mutant phenotype: as schematized in **Fig 8A**, genes that are expressed in specific subsets of neuron classes are terminal selector dependent, while genes that are expressed in a non-neuron-class–specific manner are regulated by independent means [60].

The terminal selector function of *hlh-4* is likely exerted in collaboration with the canonical AS-C cofactor, *hlh-2/*Da, which shares with *hlh-4* the unusual feature of postmitotic expression throughout the life of the ADL neuron class. *hlh-2* is also continuously expressed in a small number of additional neuron classes, but its function in these neurons remains unknown. In yeast one-hybrid assays, HLH-4/HLH-2 has been shown to bind to the CACCTG sequence that we describe here [30]. While the HLH-4/HLH2 complex and its cognate binding site is essential—and at least in some context also sufficient—for gene expression in ADL, it is unlikely to act on its own. With its 6-bp length, the recognition element of the HLH-4/HLH-2 heterodimer occurs too frequently in the genome to direct HLH-2/HLH-4 exclusively to ADL-expressed genes. We find that the LIM homeobox gene *lin-11* assists *hlh-4* in the regulation of some but not all *hlh-4*-dependent target genes. As no DNA *cis*-Regulatory motif was found to be significantly enriched in ADL-expressed genes by our bioinformatic analysis in addition to the E-box, we propose that *hlh-4* is a central core inducer of all ADL-specific genes but may be assisted in its function, i.e., provided the proper specificity, by interaction with a suite of distinct, target gene–dependent collaborating factors, such as *lin-11* and perhaps other, as yet to be discovered factors (**Fig 8B**).

Previous work on AS-C genes in worms has revealed that the AS-C-type *hlh-14* gene acts as a conventional proneural gene during early embryonic patterning to specify the neuronal identity of an AB-blastomere-derived lineage branch that produces several sensory neurons, including ADL [12]. In the absence of *hlh-14*, cells in this lineage branch convert to a hypodermal identity [12] (**Fig 8C**). Hence, the ADL neuron depends on the successive activity of two distinct AS-C-type genes, one acting as a conventional proneural gene (*hlh-14*), followed by *hlh-4*, which acts in a subbranch of this lineage, to specify terminal ADL identity (**Fig 8C**). Whether *hlh-14* directly activates *hlh-4* expression is presently unclear. Notably, though, the E-box motif in the *hlh-4* locus that is required for maintaining *hlh-4* expression is not required for initiation of *hlh-4* expression in the embryo.

Even though a proneural function of AS-C-type genes is clearly a deeply conserved function of bHLH genes, our findings prompt the intriguing question as to whether a function of bHLH genes in directly controlling the differentiated state of a neuron may have been an even more ancestral function of AS-C-type bHLH genes. In support of such notion, the AS-C ortholog in the cnidarian *Hydra magnipapillata*, *Cnash*, was previously reported to not be expressed in neuronal precursors but rather in differentiating and mature neurons, leading the authors of that report to postulate a role of hydra *Cnash* in initiating and maintaining the neuronal phenotype [64], exactly as we propose here for *C*. *elegans hlh-4*. Loss of function studies of the AS-C orthology *NvashA* of the sea anemone *Nematostella vectensis* cannot distinguish between a proneural versus terminal differentiation role [65].

Subsequent to such terminal differentiation role, an “ur-” bHLH may then have become co-opted into more upstream regulatory events in proliferating blast cells. A somewhat similar trajectory has been proposed for the Pax6/Eyeless gene, originating with a function in regulating lens protein to subsequent recruitment to earlier steps of eye development [66]. Of course, it is also conceivable that the terminal selector function of *hlh-4* may be a derived feature, one that perhaps came into existence via the acquisition of an E-box motif in the *hlh-4* locus that lead to *hlh-4* expression being “locked” into a terminal and continuous function. More detailed expression pattern analysis of AS-C and Ato-like genes in the adult nervous system of other species will provide hints whether *hlh-4-*like, terminal selector functions may also be carried by AS-C/Atonal genes in other organisms. In fact, such function may be conceivable in an already previously reported case. *Drosophila* Atonal is expressed in mature dorsal cluster neurons in the dorsolateral CNS of the flies [67]. In these neurons, Ato has no proneural function but instead serves to control arborization patterns. However, whether Ato has an impact as broad as *hlh-4* on controlling the differentiated state of these neurons is not yet known.

*C*. *elegans sox-2*/SoxB1 is another gene whose orthologs in other organisms (SoxB factors) act in early neuronal patterning [68] but that has become employed as a terminal selector in *C*. *elegans* [69,70]. Here again, the question is whether such late role is a reflection of an ancestral or derived function of this gene. It is important to keep in mind that the existence of such late functions (in addition to the well-characterized early functions) may have very easily escaped detection in other organisms, because straight knockout approaches will only reveal the early function of a gene in the lineage. Only if an early function is not existent, as apparently is the case for *sox-2* and *hlh-4*, will a late function be revealed with relative ease using standard genetic loss of function, i.e., straight knockout approaches (this paper) [69,70].

Defining *hlh-4* as a terminal selector of ADL identity sheds additional mechanistic context on previous studies about the feeding state–dependent regulation of a sensory-type GPCR gene, *srh-234*, in the ADL neuron [35,71]. Focusing on this specific gene, the authors found that the MEF-2 transcription factor, a well-known mediator of neuron activity–dependent processes in many different organisms [72], down-regulates *hlh-4-*dependent *srh-234* expression under starvation conditions. This effect is mediated via a MEF-2 binding site in the *srh-234* locus that is located next to the HLH-4/HLH-2 binding E-box [35]. Together with our description of a broad role of *hlh-4* in controlling the differentiated state of ADL, an intersectional strategy of a “genetically hardwired” identity factor with a condition-dependent factor becomes apparent. Such an intersectional strategy could perhaps be a general strategy to explain the cellular specificity of broadly acting signals that convey environmental or physiological information.

One of the remarkable features of the chemosensory system of *C*. *elegans* is the coexpression of multiple sensory receptors of the GPCR family in individual neuron types [44–46]. Even though the expression of only about one fifth of *C*. *elegans* chemosensory-type GPCRs has been examined so far [46], there are several chemosensory neurons that coexpress several dozens of GPCRs. This tremendous extent of coexpression only applies to a select set of chemosensory neurons, with the most prominent set being the nociceptive ADL, ASH, PHA, and PHB neurons [46]. One could have imagined several scenarios by which such coexpression is controlled. A previous bioinformatic analysis already strongly hinted toward coregulation of coexpressed GPCRs via a common *cis*-Regulatory motif [45]. However, it is only through the present analysis that we can conclude that a single *trans*-acting factor instructs, apparently via direct binding to a *cis*-Regulatory element shared by most if not all coexpressed GPCRs, the enormously broad spectrum of chemosensory capacities of one of these nociceptive neurons, ADL.

## Supporting information

S1 Fig Cellular expression of drivers used for *hlh-4* misexpression. **(A)** Expression of *arrd-4* promoter in all ciliated sensory neurons. **(B)** Expression of a 568-bp fragment upstream of the *unc-3* coding region fused to *rfp*, kindly provided by John Kerk. Expression is observed in cholinergic ventral cord motor neurons and presently unidentified head neurons. Whether these neurons reflect the endogenous sites of *unc-3* expression has not been determined but is irrelevant for the purpose of *hlh-4* misexpression.
(TIF)

S1 Table ADL expressed genes. Listed are all known ADL expressed genes (as per Wormbase) except genes that are either not clear terminal markers (TFs and RNP) or not neuron-type specific (pan-ciliary genes); such genes were part of the FIRE analysis but are not shown here. Bold: training dataset for original FIRE analysis. Green, non-bold: known to be expressed in ADL but not included in the training set for FIRE analysis. Blue, non-bold: gfp fusions generated in this paper. Green shade: conserved in all species that have orthologs; red shade: no motif in ortholog. The E-box motifs of *srh-132*, *srh-186*, *sri-51*, *srh-220*, *sro-1*, *hlh-2*, *nlp-7*, *nlp-10*, *osm-9*, *gpa-1*, *cam-1*, and *tax-6* sites were also bioinformatically identified in [45]. FIRE, Finding Informative Regulatory Elements; RNP, RNA binding protein; TF, transcription factor.
(XLSX)

S2 Table Top 1,000 hits from TargetOrtho search with HLH-2/HLH-4 E-box motif. (XLSX)

S3 Table Strain list. (XLSX)

S1 Data Numerical values for graphs. These datasets provide the numerical values for the graphs shown in Fig 2, Fig 3, Fig 4 and Fig 7.
(XLSX)

## Abbreviations

AS-C: Achaete-Scute complex
bHLH: basic helix-loop-helix
CNS: central nervous system
Da: Daughterless
FIRE: Finding Informative Regulatory Elements
GFP: green fluorescent protein
GPCR: G-protein coupled receptor
RFP: red fluorescent protein
VGLUT: vesicular glutamate transporter

## References

[1] Bertrand N, Castro DS, Guillemot F. Proneural genes and the specification of neural cell types. Nat Rev Neurosci. 2002;3(7):517–30. doi: 10.1038/nrn874 . 12094208 10.1038/nrn874

[2] Jan YN, Jan LY. Neuronal cell fate specification in Drosophila. Curr Opin Neurobiol. 1994;4(1):8–13. . 8173329 10.1016/0959-4388(94)90025-6

[3] Campuzano S, Modolell J. Patterning of the Drosophila nervous system: the achaete-scute gene complex. Trends Genet. 1992;8(6):202–8. . 1496555 10.1016/0168-9525(92)90234-u

[4] Jarman AP, Grau Y, Jan LY, Jan YN. atonal is a proneural gene that directs chordotonal organ formation in the Drosophila peripheral nervous system. Cell. 1993;73(7):1307–21. . 8324823 10.1016/0092-8674(93)90358-w

[5] Villares R, Cabrera CV. The achaete-scute gene complex of D. melanogaster: conserved domains in a subset of genes required for neurogenesis and their homology to myc. Cell. 1987;50(3):415–24. . 3111716 10.1016/0092-8674(87)90495-8

[6] Hassan BA, Bellen HJ. Doing the MATH: is the mouse a good model for fly development? Genes Dev. 2000;14(15):1852–65. . 10921900

[7] Ben-Arie N, Bellen HJ, Armstrong DL, McCall AE, Gordadze PR, Guo Q, et al Math1 is essential for genesis of cerebellar granule neurons. Nature. 1997;390(6656):169–72. doi: 10.1038/36579 . 9367153 10.1038/36579

[8] Guillemot F. Analysis of the role of basic-helix-loop-helix transcription factors in the development of neural lineages in the mouse. Biol Cell. 1995;84(1–2):3–6. . 8574196 10.1016/0248-4900(96)81312-8

[9] Ledent V, Vervoort M. The basic helix-loop-helix protein family: comparative genomics and phylogenetic analysis. Genome Res. 2001;11(5):754–70. doi: 10.1101/gr.177001 11337472 10.1101/gr.177001 PMC311049

[10] Zhao C, Emmons SW. A transcription factor controlling development of peripheral sense organs in C. elegans. Nature. 1995;373(6509):74–8. doi: 10.1038/373074a0 7800042 10.1038/373074a0

[11] Frank CA, Baum PD, Garriga G. HLH-14 is a C. elegans achaete-scute protein that promotes neurogenesis through asymmetric cell division. Development. 2003;130(26):6507–18. Epub 2003/11/25. doi: 10.1242/dev.00894 [pii]. . 14627726 10.1242/dev.00894

[12] Poole RJ, Bashllari E, Cochella L, Flowers EB, Hobert O. A Genome-Wide RNAi Screen for Factors Involved in Neuronal Specification in Caenorhabditis elegans. PLoS Genet. 2011;7(6):e1002109 doi: 10.1371/journal.pgen.1002109 ; PubMed Central PMCID: PMC3116913. 21698137 10.1371/journal.pgen.1002109 PMC3116913

[13] Guillemot F, Hassan BA. Beyond proneural: emerging functions and regulations of proneural proteins. Curr Opin Neurobiol. 2017;42:93–101. doi: 10.1016/j.conb.2016.11.011 . 28025176 10.1016/j.conb.2016.11.011

[14] Portman DS, Emmons SW. The basic helix-loop-helix transcription factors LIN-32 and HLH-2 function together in multiple steps of a C. elegans neuronal sublineage. Development. 2000;127(24):5415–26. 11076762 10.1242/dev.127.24.5415

[15] Brenner S. The genetics of Caenorhabditis elegans. Genetics. 1974;77(1):71–94. 4366476 10.1093/genetics/77.1.71 PMC1213120

[16] Bond SR, Naus CC. RF-Cloning.org: an online tool for the design of restriction-free cloning projects. Nucleic Acids Res. 2012;40(Web Server issue):W209–13. doi: 10.1093/nar/gks396 ; PubMed Central PMCID: PMCPMC3394257. 22570410 10.1093/nar/gks396 PMC3394257

[17] Hobert O. PCR fusion-based approach to create reporter gene constructs for expression analysis in transgenic C. elegans. BioTechniques. 2002;32(4):728–30. . 11962590 10.2144/02324bm01

[18] Patel T, Tursun B, Rahe DP, Hobert O. Removal of Polycomb Repressive Complex 2 Makes C. elegans Germ Cells Susceptible to Direct Conversion into Specific Somatic Cell Types. Cell Rep. 2012;2(5):1178–86. Epub 2012/10/30. doi: 10.1016/j.celrep.2012.09.020 [pii]. . 23103163 10.1016/j.celrep.2012.09.020 PMC3529301

[19] Sarov M, Murray JI, Schanze K, Pozniakovski A, Niu W, Angermann K, et al A genome-scale resource for in vivo tag-based protein function exploration in C. elegans. Cell. 2012;150(4):855–66. doi: 10.1016/j.cell.2012.08.001 ; PubMed Central PMCID: PMC3979301. 22901814 10.1016/j.cell.2012.08.001 PMC3979301

[20] Tursun B, Cochella L, Carrera I, Hobert O. A toolkit and robust pipeline for the generation of fosmid-based reporter genes in C. elegans. PLoS ONE. 2009;4(3):e4625 doi: 10.1371/journal.pone.0004625 . 19259264 10.1371/journal.pone.0004625 PMC2649505

[21] Schindelin J, Arganda-Carreras I, Frise E, Kaynig V, Longair M, Pietzsch T, et al Fiji: an open-source platform for biological-image analysis. Nat Methods. 2012;9(7):676–82. doi: 10.1038/nmeth.2019 ; PubMed Central PMCID: PMCPMC3855844. 22743772 10.1038/nmeth.2019 PMC3855844

[22] Schnabel R, Hutter H, Moerman D, Schnabel H. Assessing normal embryogenesis in Caenorhabditis elegans using a 4D microscope: variability of development and regional specification. Dev Biol. 1997;184(2):234–65. doi: 10.1006/dbio.1997.8509 9133433 10.1006/dbio.1997.8509

[23] Hilliard MA, Bargmann CI, Bazzicalupo P. C. elegans responds to chemical repellents by integrating sensory inputs from the head and the tail. Curr Biol. 2002;12(9):730–4. . 12007416 10.1016/s0960-9822(02)00813-8

[24] Jang H, Kim K, Neal SJ, Macosko E, Kim D, Butcher RA, et al Neuromodulatory state and sex specify alternative behaviors through antagonistic synaptic pathways in C. elegans. Neuron. 2012;75(4):585–92. doi: 10.1016/j.neuron.2012.06.034 ; PubMed Central PMCID: PMC3462069. 22920251 10.1016/j.neuron.2012.06.034 PMC3462069

[25] Ibsen S, Tong A, Schutt C, Esener S, Chalasani SH. Sonogenetics is a non-invasive approach to activating neurons in Caenorhabditis elegans. Nat Commun. 2015;6:8264 doi: 10.1038/ncomms9264 ; PubMed Central PMCID: PMC4571289. 26372413 10.1038/ncomms9264 PMC4571289

[26] Elemento O, Slonim N, Tavazoie S. A universal framework for regulatory element discovery across all genomes and data types. Mol Cell. 2007;28(2):337–50. doi: 10.1016/j.molcel.2007.09.027 ; PubMed Central PMCID: PMC2900317. 17964271 10.1016/j.molcel.2007.09.027 PMC2900317

[27] Glenwinkel L, Wu D, Minevich G, Hobert O. TargetOrtho: a phylogenetic footprinting tool to identify transcription factor targets. Genetics. 2014;197(1):61–76. doi: 10.1534/genetics.113.160721 ; PubMed Central PMCID: PMC4012501. 24558259 10.1534/genetics.113.160721 PMC4012501

[28] Sulston JE, Schierenberg E, White JG, Thomson JN. The embryonic cell lineage of the nematode Caenorhabditis elegans. Dev Biol. 1983;100(1):64–119. 6684600 10.1016/0012-1606(83)90201-4

[29] Krause M, Park M, Zhang JM, Yuan J, Harfe B, Xu SQ, et al A C. elegans E/Daughterless bHLH protein marks neuronal but not striated muscle development. Development. 1997;124(11):2179–89. . 9187144 10.1242/dev.124.11.2179

[30] Grove CA, De Masi F, Barrasa MI, Newburger DE, Alkema MJ, Bulyk ML, et al A multiparameter network reveals extensive divergence between C. elegans bHLH transcription factors. Cell. 2009;138(2):314–27. doi: 10.1016/j.cell.2009.04.058 ; PubMed Central PMCID: PMC2774807. 19632181 10.1016/j.cell.2009.04.058 PMC2774807

[31] Doonan R, Hatzold J, Raut S, Conradt B, Alfonso A. HLH-3 is a C. elegans Achaete/Scute protein required for differentiation of the hermaphrodite-specific motor neurons. Mech Dev. 2008;125(9–10):883–93. Epub 2008/07/01. doi: S0925-4773(08)00079-8 [pii] doi: 10.1016/j.mod.2008.06.002 . 18586090 10.1016/j.mod.2008.06.002

[32] Smit RB, Schnabel R, Gaudet J. The HLH-6 transcription factor regulates C. elegans pharyngeal gland development and function. PLoS Genet. 2008;4(10):e1000222 doi: 10.1371/journal.pgen.1000222 ; PubMed Central PMCID: PMC2563036. 18927627 10.1371/journal.pgen.1000222 PMC2563036

[33] Mathies LD, Henderson ST, Kimble J. The C. elegans Hand gene controls embryogenesis and early gonadogenesis. Development. 2003;130(13):2881–92. . 12756172 10.1242/dev.00483

[34] Tamai KK, Nishiwaki K. bHLH transcription factors regulate organ morphogenesis via activation of an ADAMTS protease in C. elegans. Dev Biol. 2007;308(2):562–71. doi: 10.1016/j.ydbio.2007.05.024 . 17588558 10.1016/j.ydbio.2007.05.024

[35] Gruner M, Grubbs J, McDonagh A, Valdes D, Winbush A, van der Linden AM. Cell-Autonomous and Non-Cell-Autonomous Regulation of a Feeding State-Dependent Chemoreceptor Gene via MEF-2 and bHLH Transcription Factors. PLoS Genet. 2016;12(8):e1006237 doi: 10.1371/journal.pgen.1006237 ; PubMed Central PMCID: PMC4972359. 27487365 10.1371/journal.pgen.1006237 PMC4972359

[36] Way JC, Chalfie M. The mec-3 gene of Caenorhabditis elegans requires its own product for maintained expression and is expressed in three neuronal cell types. Genes Dev. 1989;3(12A):1823–33. 2576011 10.1101/gad.3.12a.1823

[37] Baumeister R, Liu Y, Ruvkun G. Lineage-specific regulators couple cell lineage asymmetry to the transcription of the Caenorhabditis elegans POU gene unc-86 during neurogenesis. Genes Dev. 1996;10(11):1395–410. Epub 1996/06/01. . 8647436 10.1101/gad.10.11.1395

[38] Hobert O, Mori I, Yamashita Y, Honda H, Ohshima Y, Liu Y, et al Regulation of interneuron function in the C. elegans thermoregulatory pathway by the ttx-3 LIM homeobox gene. Neuron. 1997;19(2):345–57. 9292724 10.1016/s0896-6273(00)80944-7

[39] Etchberger JF, Lorch A, Sleumer MC, Zapf R, Jones SJ, Marra MA, et al The molecular signature and cis-regulatory architecture of a C. elegans gustatory neuron. Genes Dev. 2007;21(13):1653–74. doi: 10.1101/gad.1560107 . 17606643 10.1101/gad.1560107 PMC1899474

[40] Consortium CeDM. large-scale screening for targeted knockouts in the Caenorhabditis elegans genome. G3 (Bethesda). 2012;2(11):1415–25. doi: 10.1534/g3.112.003830 ; PubMed Central PMCID: PMCPMC3484672. 23173093 10.1534/g3.112.003830 PMC3484672

[41] Perkins LA, Hedgecock EM, Thomson JN, Culotti JG. Mutant sensory cilia in the nematode Caenorhabditis elegans. Dev Biol. 1986;117(2):456–87. 2428682 10.1016/0012-1606(86)90314-3

[42] Collet J, Spike CA, Lundquist EA, Shaw JE, Herman RK. Analysis of osm-6, a gene that affects sensory cilium structure and sensory neuron function in Caenorhabditis elegans. Genetics. 1998;148(1):187–200. . 9475731 10.1093/genetics/148.1.187 PMC1459801

[43] Blacque OE, Perens EA, Boroevich KA, Inglis PN, Li C, Warner A, et al Functional genomics of the cilium, a sensory organelle. Curr Biol. 2005;15(10):935–41. doi: 10.1016/j.cub.2005.04.059 . 15916950 10.1016/j.cub.2005.04.059

[44] Troemel ER, Chou JH, Dwyer ND, Colbert HA, Bargmann CI. Divergent seven transmembrane receptors are candidate chemosensory receptors in C. elegans. Cell. 1995;83(2):207–18. 7585938 10.1016/0092-8674(95)90162-0

[45] McCarroll SA, Li H, Bargmann CI. Identification of transcriptional regulatory elements in chemosensory receptor genes by probabilistic segmentation. Curr Biol. 2005;15(4):347–52. doi: 10.1016/j.cub.2005.02.023 . 15723796 10.1016/j.cub.2005.02.023

[46] Vidal B, Aghayeva U, Sun H, Wang C, Glenwinkel L, Bayer EA, et al An atlas of Caenorhabditis elegans chemoreceptor expression. PLoS Biol. 2018;16(1):e2004218 doi: 10.1371/journal.pbio.2004218 ; PubMed Central PMCID: PMC5749674. 29293491 10.1371/journal.pbio.2004218 PMC5749674

[47] Hilliard MA, Apicella AJ, Kerr R, Suzuki H, Bazzicalupo P, Schafer WR. In vivo imaging of C. elegans ASH neurons: cellular response and adaptation to chemical repellents. EMBO J. 2005;24(1):63–72. doi: 10.1038/sj.emboj.7600493 ; PubMed Central PMCID: PMCPMC544906. 15577941 10.1038/sj.emboj.7600493 PMC544906

[48] Rogers C, Persson A, Cheung B, de Bono M. Behavioral motifs and neural pathways coordinating O2 responses and aggregation in C. elegans. Curr Biol. 2006;16(7):649–59. Epub 2006/04/04. doi: S0960-9822(06)01316-9 [pii] doi: 10.1016/j.cub.2006.03.023 . 16581509 10.1016/j.cub.2006.03.023

[49] Colbert HA, Smith TL, Bargmann CI. OSM-9, a novel protein with structural similarity to channels, is required for olfaction, mechanosensation, and olfactory adaptation in Caenorhabditis elegans. J Neurosci. 1997;17(21):8259–69. 9334401 10.1523/JNEUROSCI.17-21-08259.1997 PMC6573730

[50] White JG, Southgate E, Thomson JN, Brenner S. The structure of the nervous system of the nematode *Caenorhabditis elegans*. Philosophical Transactions of the Royal Society of London B Biological Sciences. 1986;314:1–340. 22462104 10.1098/rstb.1986.0056

[51] Serrano-Saiz E, Poole RJ, Felton T, Zhang F, de la Cruz ED, Hobert O. Modular Control of Glutamatergic Neuronal Identity in C. elegans by Distinct Homeodomain Proteins. Cell. 2013;155:659–73. doi: 10.1016/j.cell.2013.09.052 24243022 10.1016/j.cell.2013.09.052 PMC3855022

[52] Kim K, Li C. Expression and regulation of an FMRFamide-related neuropeptide gene family in Caenorhabditis elegans. The Journal of comparative neurology. 2004;475(4):540–50. doi: 10.1002/cne.20189 . 15236235 10.1002/cne.20189

[53] Nathoo AN, Moeller RA, Westlund BA, Hart AC. Identification of neuropeptide-like protein gene families in Caenorhabditiselegans and other species. Proc Natl Acad Sci U S A. 2001;98(24):14000–5. doi: 10.1073/pnas.241231298 . 11717458 10.1073/pnas.241231298 PMC61156

[54] Hall DH. Gap junctions in C. elegans: Their roles in behavior and development. Dev Neurobiol. 2017;77(5):587–96. doi: 10.1002/dneu.22408 ; PubMed Central PMCID: PMC5412865. 27294317 10.1002/dneu.22408 PMC5412865

[55] Altun ZF, Chen B, Wang ZW, Hall DH. High resolution map of Caenorhabditis elegans gap junction proteins. Dev Dyn. 2009;238(8):1936–50. Epub 2009/07/22. doi: 10.1002/dvdy.22025 ; PubMed Central PMCID: PMC2732576. 19621339 10.1002/dvdy.22025 PMC2732576

[56] Sharma V, He C, Sacca-Schaeffer J, Brzozowski E, Martin-Herranz DE, Mendelowitz Z, et al Insight into the family of Na+/Ca2+ exchangers of Caenorhabditis elegans. Genetics. 2013;195(2):611–9. doi: 10.1534/genetics.113.153106 ; PubMed Central PMCID: PMC3781985. 23893482 10.1534/genetics.113.153106 PMC3781985

[57] Kunitomo H, Uesugi H, Kohara Y, Iino Y. Identification of ciliated sensory neuron-expressed genes in Caenorhabditis elegans using targeted pull-down of poly(A) tails. Genome Biol. 2005;6(2):R17 doi: 10.1186/gb-2005-6-2-r17 ; PubMed Central PMCID: PMCPMC551537. 15693946 10.1186/gb-2005-6-2-r17 PMC551537

[58] Halder G, Callaerts P, Gehring WJ. Induction of ectopic eyes by targeted expression of the eyeless gene in Drosophila [see comments]. Science. 1995;267(5205):1788–92. 7892602 10.1126/science.7892602

[59] Massari ME, Murre C. Helix-loop-helix proteins: regulators of transcription in eucaryotic organisms. Mol Cell Biol. 2000;20(2):429–40. ; PubMed Central PMCID: PMC85097. 10611221 10.1128/mcb.20.2.429-440.2000 PMC85097

[60] Stefanakis N, Carrera I, Hobert O. Regulatory Logic of Pan-Neuronal Gene Expression in C. elegans. Neuron. 2015;87(4):733–50. doi: 10.1016/j.neuron.2015.07.031 ; PubMed Central PMCID: PMC4545498. 26291158 10.1016/j.neuron.2015.07.031 PMC4545498

[61] Hobert O, D'Alberti T, Liu Y, Ruvkun G. Control of neural development and function in a thermoregulatory network by the LIM homeobox gene lin-11. J Neurosci. 1998;18(6):2084–96. 9482795 10.1523/JNEUROSCI.18-06-02084.1998 PMC6792926

[62] Hobert O. Regulatory logic of neuronal diversity: terminal selector genes and selector motifs. Proc Natl Acad Sci U S A. 2008;105(51):20067–71. Epub 2008/12/24. doi: 0806070105 [pii] doi: 10.1073/pnas.0806070105 . 19104055 10.1073/pnas.0806070105 PMC2629285

[63] Hobert O. Terminal Selectors of Neuronal Identity. Curr Top Dev Biol. 2016;116:455–75. doi: 10.1016/bs.ctdb.2015.12.007 . 26970634 10.1016/bs.ctdb.2015.12.007

[64] Hayakawa E, Fujisawa C, Fujisawa T. Involvement of Hydra achaete-scute gene CnASH in the differentiation pathway of sensory neurons in the tentacles. Dev Genes Evol. 2004;214(10):486–92. doi: 10.1007/s00427-004-0430-4 . 15378362 10.1007/s00427-004-0430-4

[65] Layden MJ, Boekhout M, Martindale MQ. Nematostella vectensis achaete-scute homolog NvashA regulates embryonic ectodermal neurogenesis and represents an ancient component of the metazoan neural specification pathway. Development. 2012;139(5):1013–22. doi: 10.1242/dev.073221 ; PubMed Central PMCID: PMCPMC3274362. 22318631 10.1242/dev.073221 PMC3274362

[66] Gehring WJ. New perspectives on eye development and the evolution of eyes and photoreceptors. J Hered. 2005;96(3):171–84. doi: 10.1093/jhered/esi027 . 15653558 10.1093/jhered/esi027

[67] Hassan BA, Bermingham NA, He Y, Sun Y, Jan YN, Zoghbi HY, et al atonal regulates neurite arborization but does not act as a proneural gene in the Drosophila brain. Neuron. 2000;25(3):549–61. . 10774724 10.1016/s0896-6273(00)81059-4

[68] Reiprich S, Wegner M. From CNS stem cells to neurons and glia: Sox for everyone. Cell Tissue Res. 2015;359(1):111–24. doi: 10.1007/s00441-014-1909-6 . 24894327 10.1007/s00441-014-1909-6

[69] Vidal B, Santella A, Serrano-Saiz E, Bao Z, Chuang CF, Hobert O. C. elegans SoxB genes are dispensable for embryonic neurogenesis but required for terminal differentiation of specific neuron types. Development. 2015;142(14):2464–77. doi: 10.1242/dev.125740 ; PubMed Central PMCID: PMC4510870. 26153233 10.1242/dev.125740 PMC4510870

[70] Alqadah A, Hsieh YW, Vidal B, Chang C, Hobert O, Chuang CF. Postmitotic diversification of olfactory neuron types is mediated by differential activities of the HMG-box transcription factor SOX-2. EMBO J. 2015;34(20):2574–89. doi: 10.15252/embj.201592188 ; PubMed Central PMCID: PMC4609187. 26341465 10.15252/embj.201592188 PMC4609187

[71] Gruner M, Nelson D, Winbush A, Hintz R, Ryu L, Chung SH, et al Feeding state, insulin and NPR-1 modulate chemoreceptor gene expression via integration of sensory and circuit inputs. PLoS Genet. 2014;10(10):e1004707 doi: 10.1371/journal.pgen.1004707 ; PubMed Central PMCID: PMC4214617. 25357003 10.1371/journal.pgen.1004707 PMC4214617

[72] Flavell SW, Greenberg ME. Signaling mechanisms linking neuronal activity to gene expression and plasticity of the nervous system. Annu Rev Neurosci. 2008;31:563–90. doi: 10.1146/annurev.neuro.31.060407.125631 ; PubMed Central PMCID: PMC2728073. 18558867 10.1146/annurev.neuro.31.060407.125631 PMC2728073

