# Supplementary figures and images for: Unconventional function of an Achaete-Scute homolog as a terminal selector of nociceptive neuron identity
Source: PLoS Biol. 2018 Apr 19;16(4):e2004979. doi: 10.1371/journal.pbio.2004979 (PMC5908064; doi:10.1371/journal.pbio.2004979)

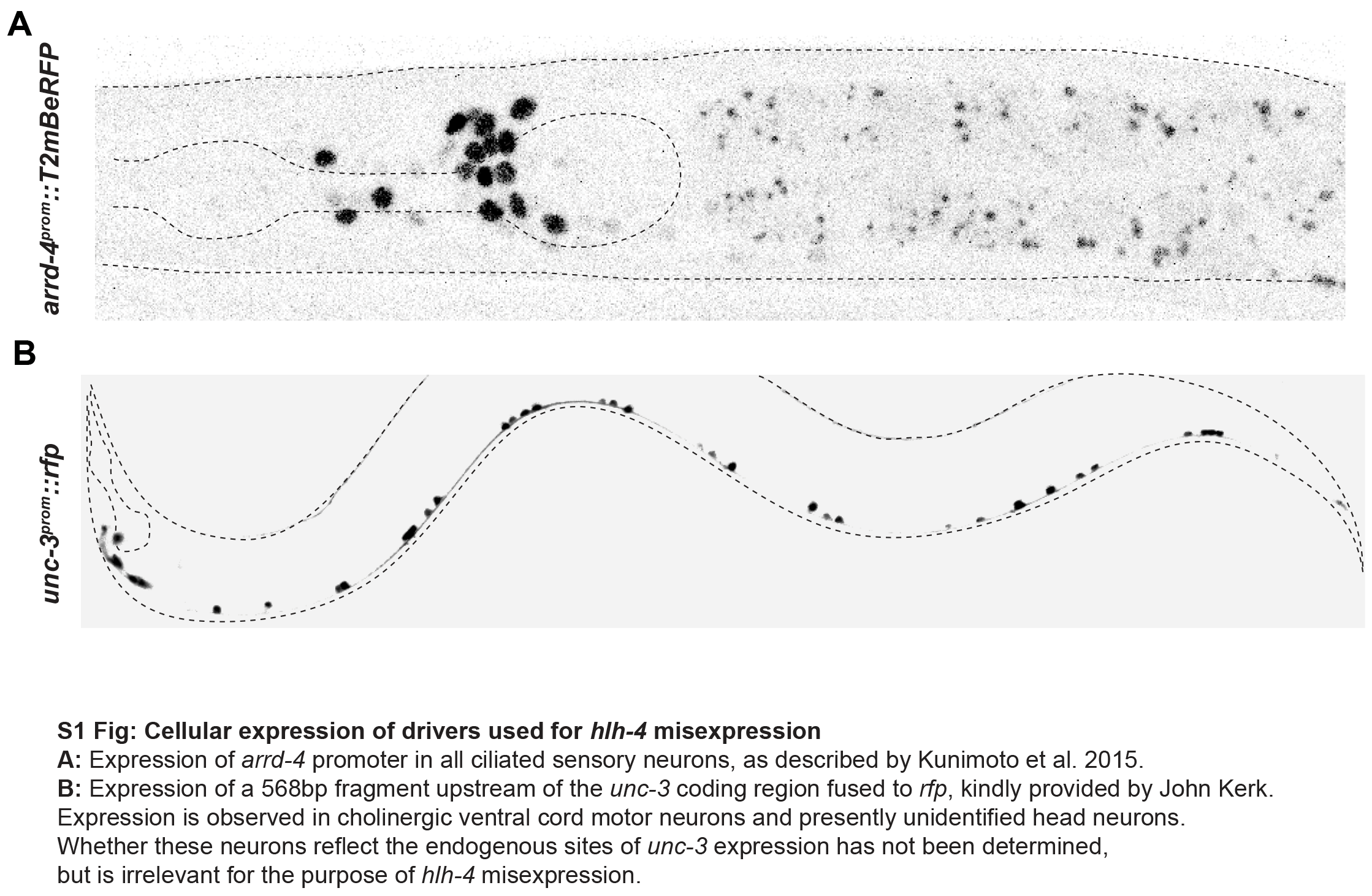

Supplement: S1 Fig — (A) Expression of arrd-4 promoter in all ciliated sensory neurons. (B) Expression of a 568-bp fragment upstream of the unc-3 coding region fused to rfp, kindly provided by John Kerk. Expression is observed in cholinergic ventral cord motor neurons and presently unidentified head neurons. Whether these neurons reflect the endogenous sites of unc-3 expression has not been determined but is irrelevant for the purpose of hlh-4 misexpression. (TIF) [file pbio.2004979.s001.tif]
